# Correction: StanDep: Capturing transcriptomic variability improves context-specific metabolic models

**DOI:** 10.1371/journal.pcbi.1009776

**Published:** 2022-01-10

**Authors:** Chintan J. Joshi, Song-Min Schinn, Anne Richelle, Isaac Shamie, Eyleen J. O’Rourke, Nathan E. Lewis

Nathan E. Lewis is incorrectly listed as the sole corresponding author for this article. Eyleen J. O’Rourke is also a corresponding author. Eyleen J. O’Rourke’s email address is: ejo8b@virginia.edu
