# Supplementary figures and images for: StanDep: Capturing transcriptomic variability improves context-specific metabolic models
Source: PLoS Comput Biol. 2020 May 12;16(5):e1007764. doi: 10.1371/journal.pcbi.1007764 (PMC7244210; doi:10.1371/journal.pcbi.1007764)

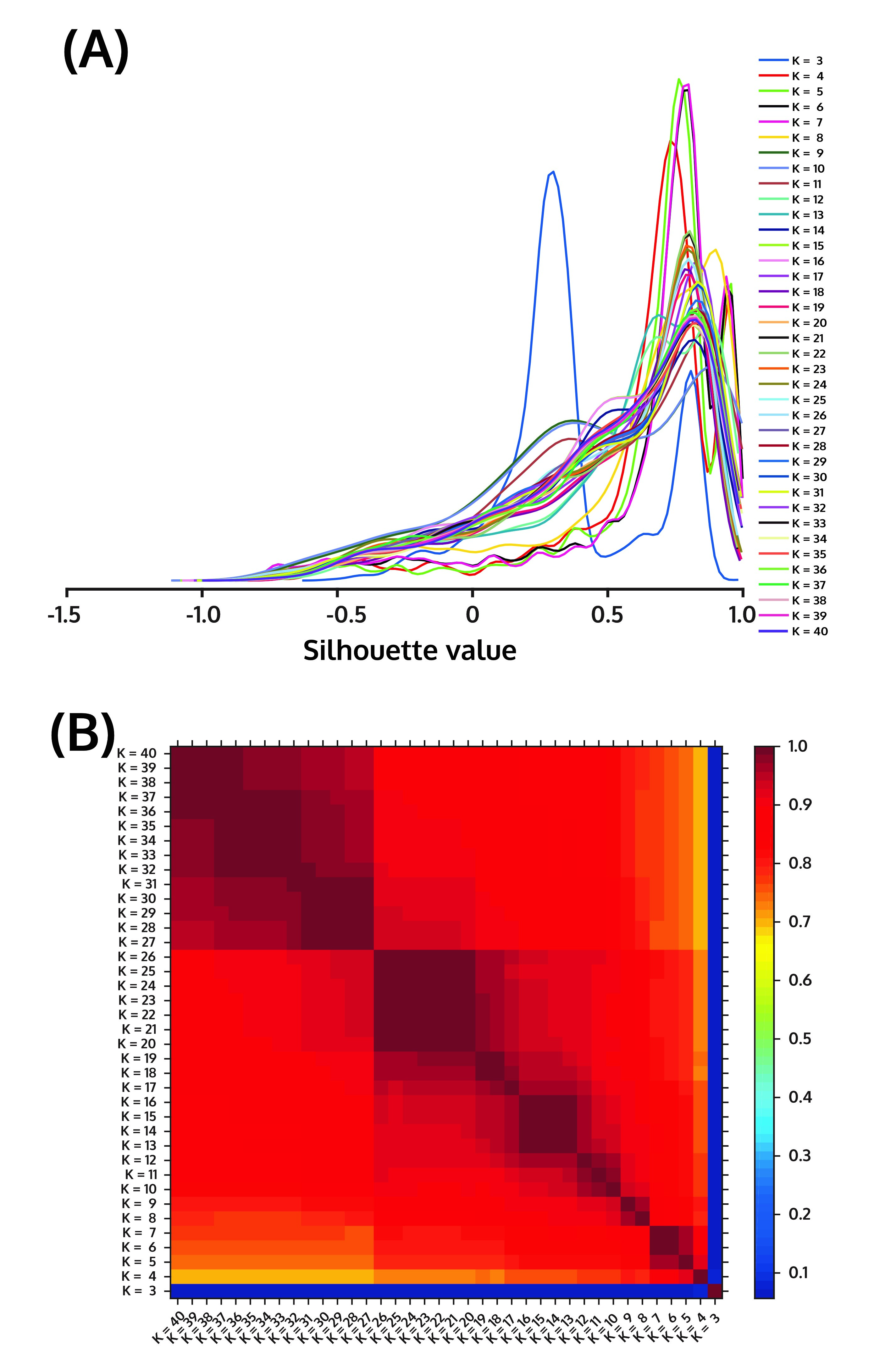

Supplement: S1 Fig — Comparison of Jaccard similarity for core reaction list of 44 cancer cell lines using (B) different number of clusters and (A) silhouette value for quality of clusters when using StanDep. The complete linkage method and Euclidean distance were used. More than 10 clusters lead to over 90% mean Jaccard similarity. (JPG) [file pcbi.1007764.s004.jpg]

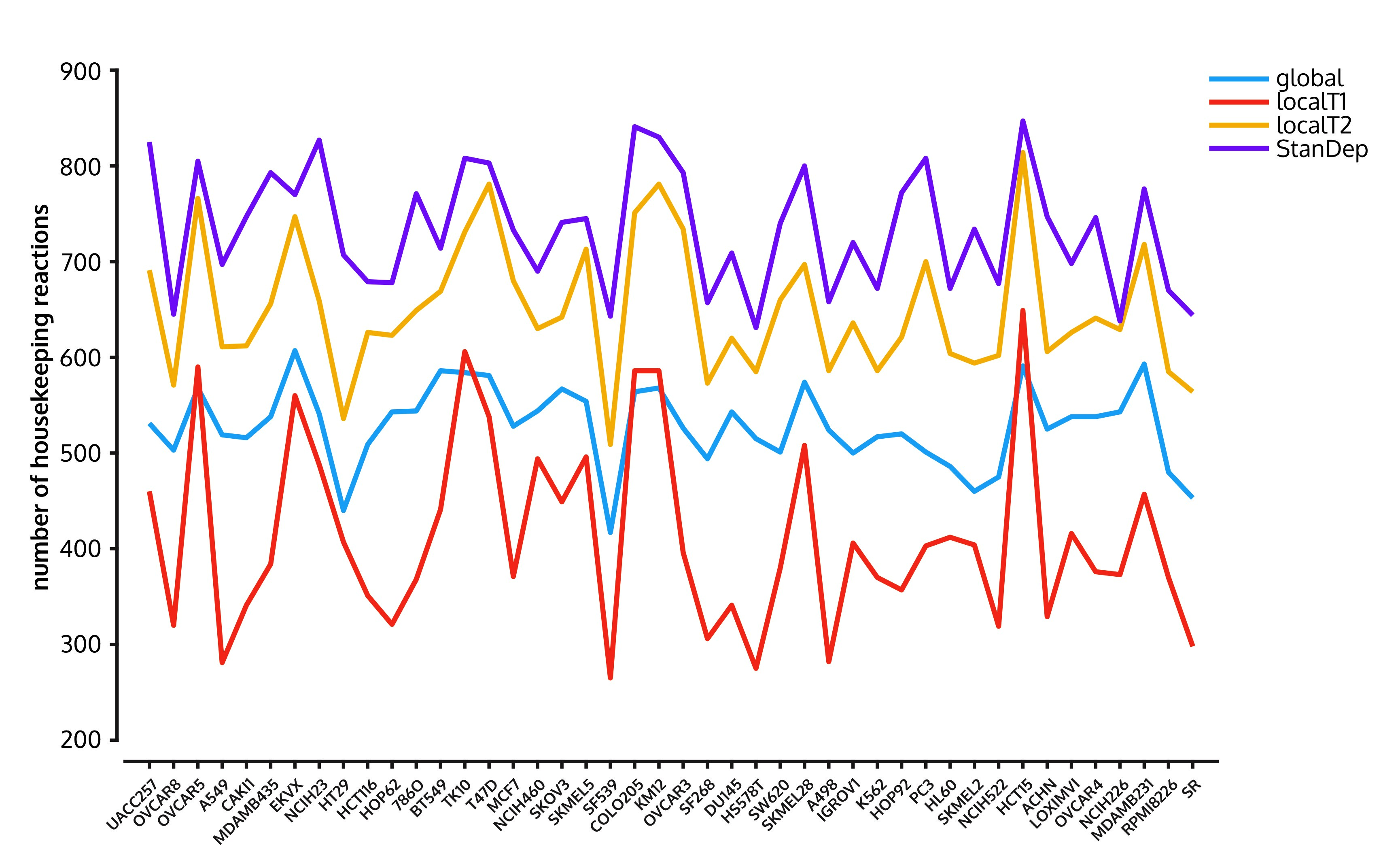

Supplement: S2 Fig — (JPG) [file pcbi.1007764.s005.jpg]

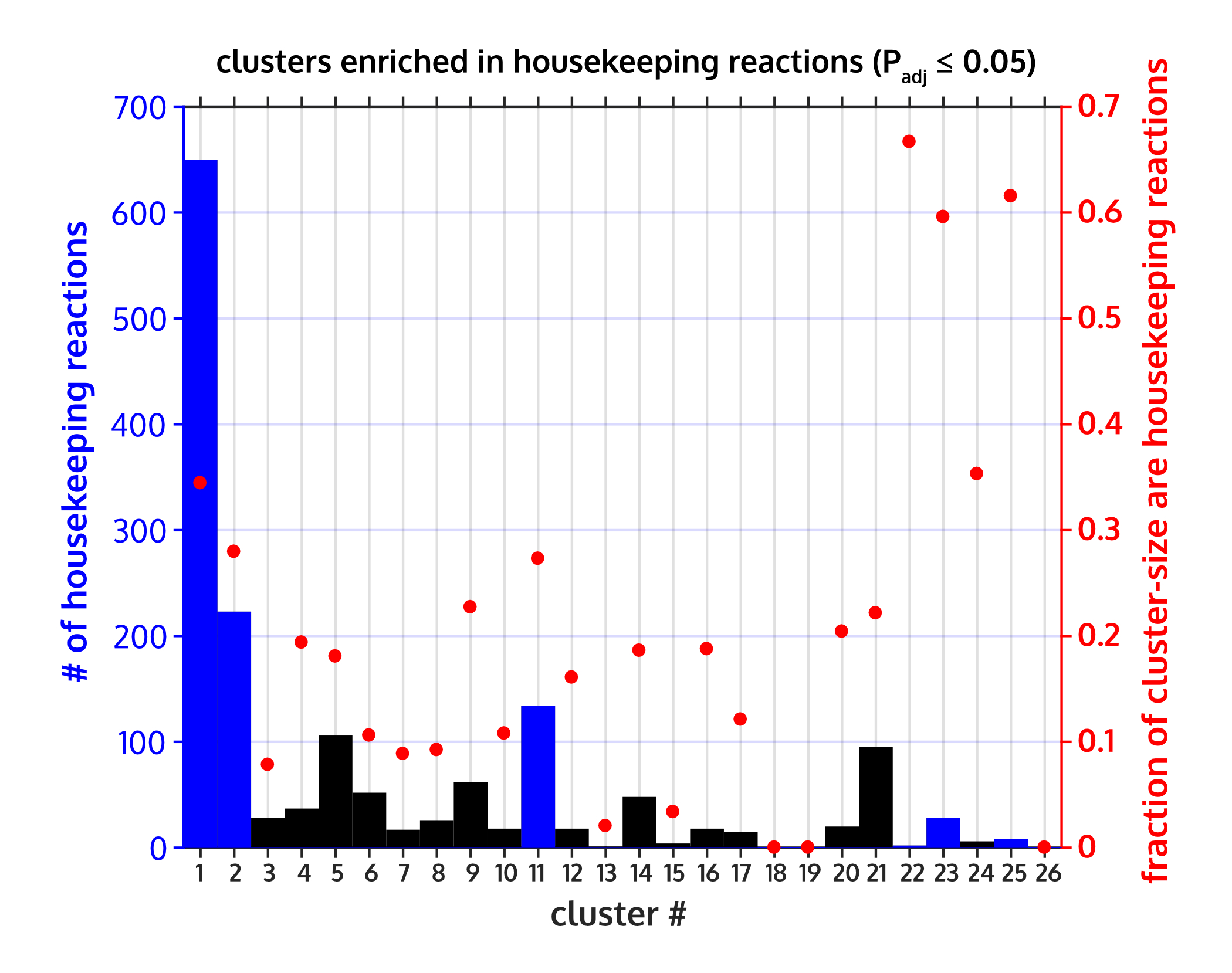

Supplement: S3 Fig — Clusters are enriched in housekeeping reactions. Enriched clusters are shown in blue bars (left axis). Clusters containing housekeeping reactions but are not enriched are shown in black bars (left axis). The fraction of reactions in each cluster that are housekeeping reactions are given in red dots (right axis). BHFDR correction was used for hypergeometric test for over-representation. (JPG) [file pcbi.1007764.s006.jpg]

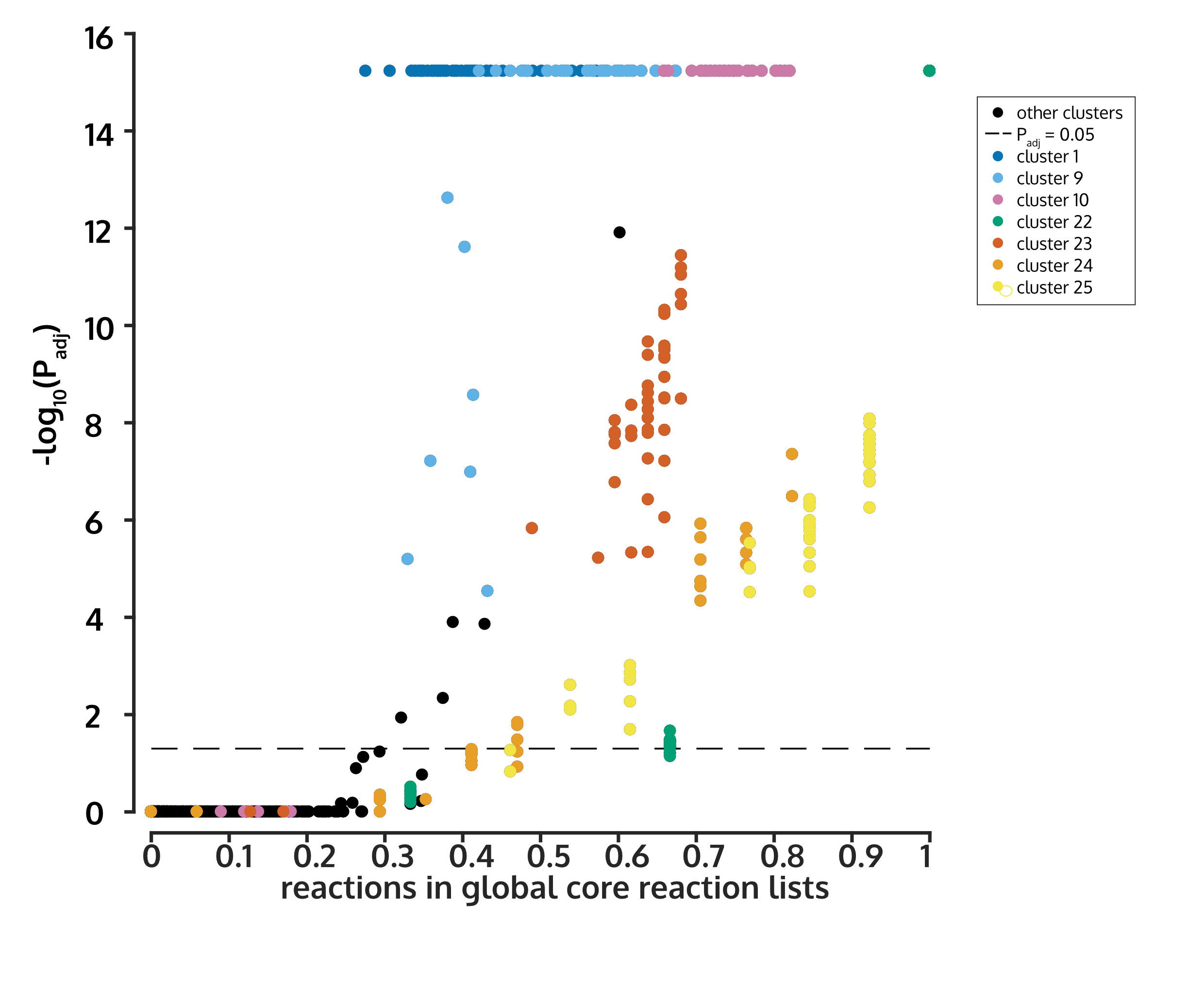

Supplement: S4 Fig — Each dot is a cell line-cluster pair. (JPG) [file pcbi.1007764.s007.jpg]

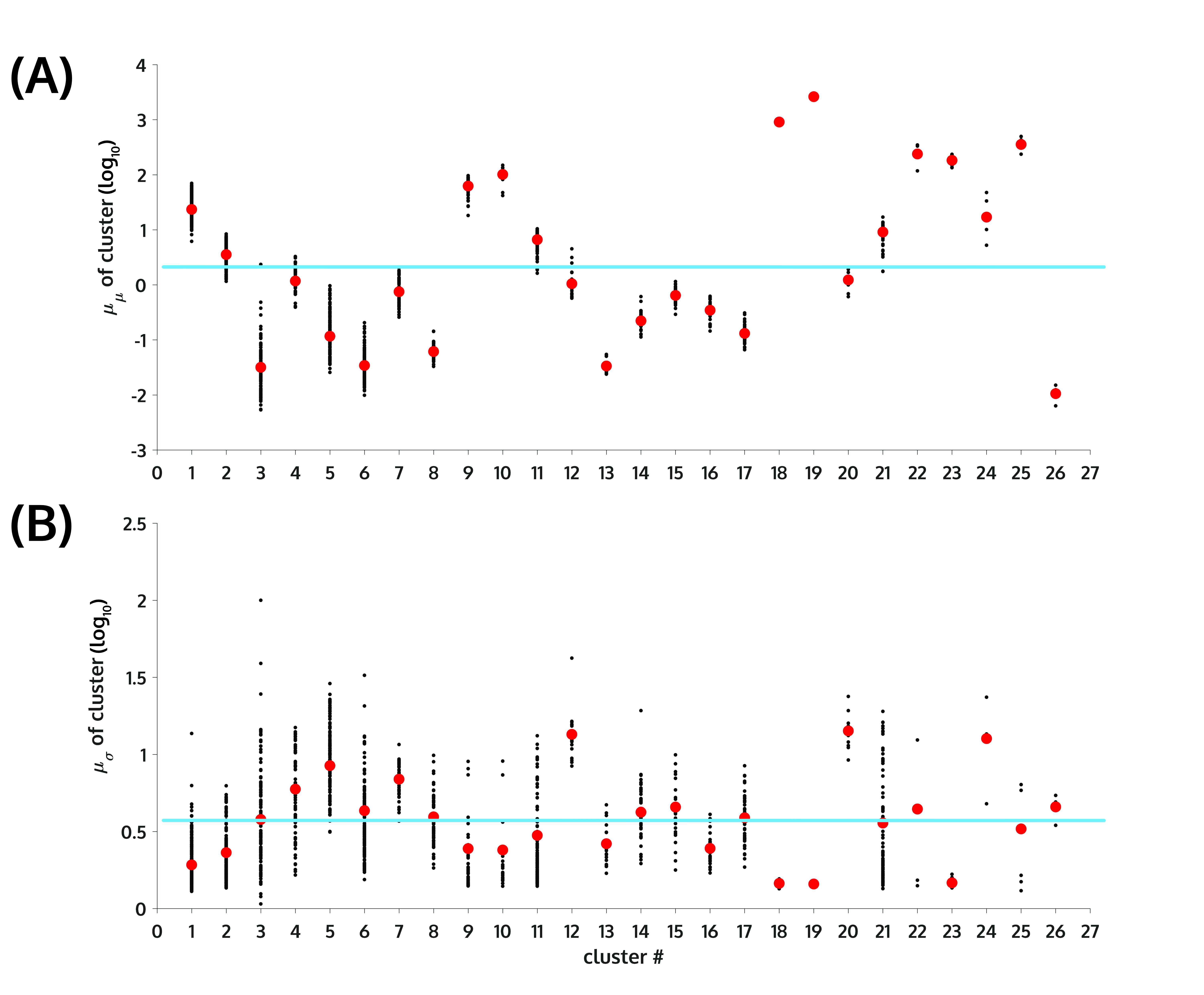

Supplement: S5 Fig — Mean (A) and standard deviation (A) of expression of each enzyme (black dots) and that of the clusters (red dots) is shown. The blue lines are the same for the entire data. (JPG) [file pcbi.1007764.s008.jpg]

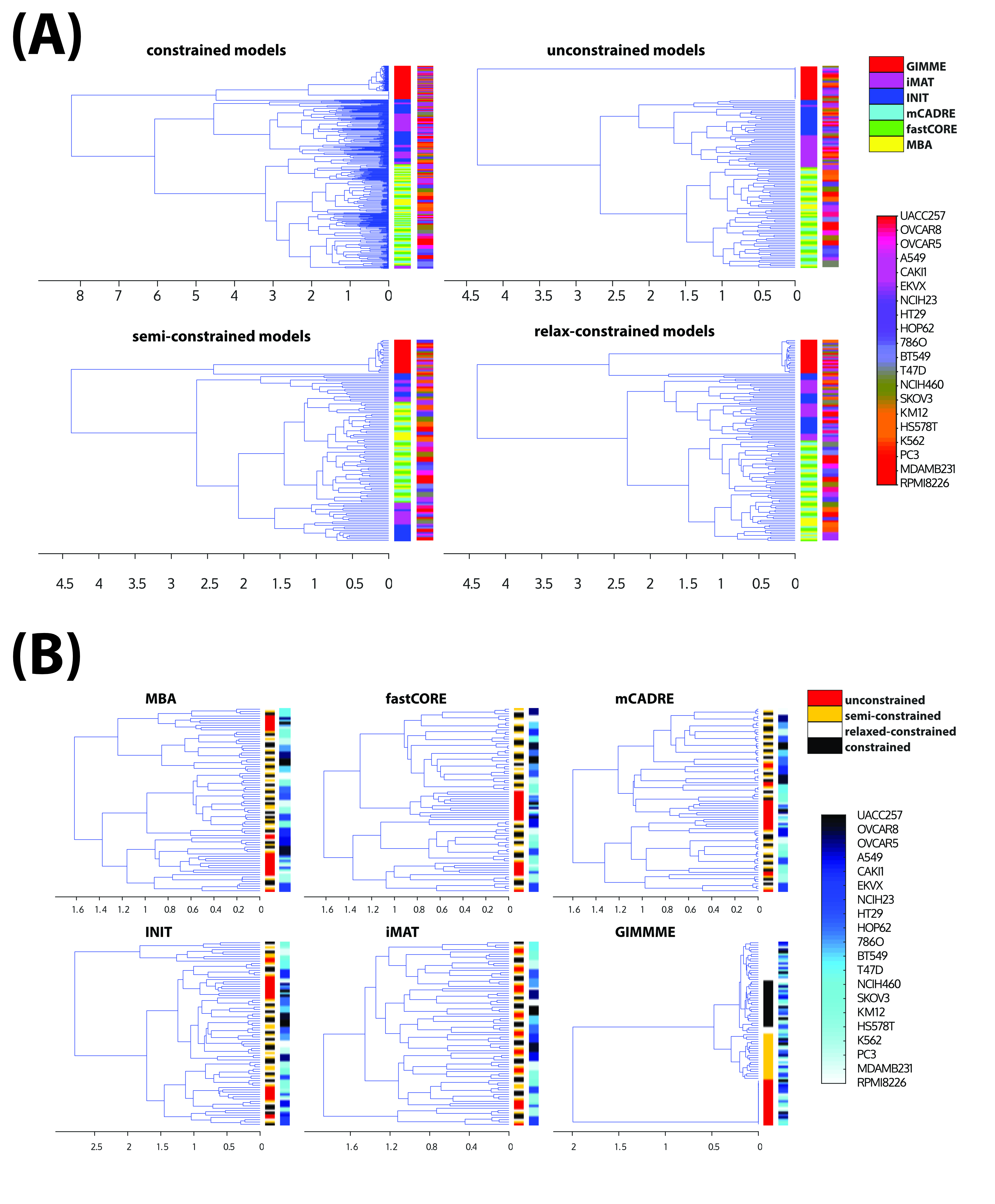

Supplement: S6 Fig — (A) Distribution of Jaccard similarity between models built using different constraint types, extraction methods, and belonging to different cell lines. (B) Distribution of Jaccard similarity between models extracted using different extraction methods. (JPG) [file pcbi.1007764.s009.jpg]

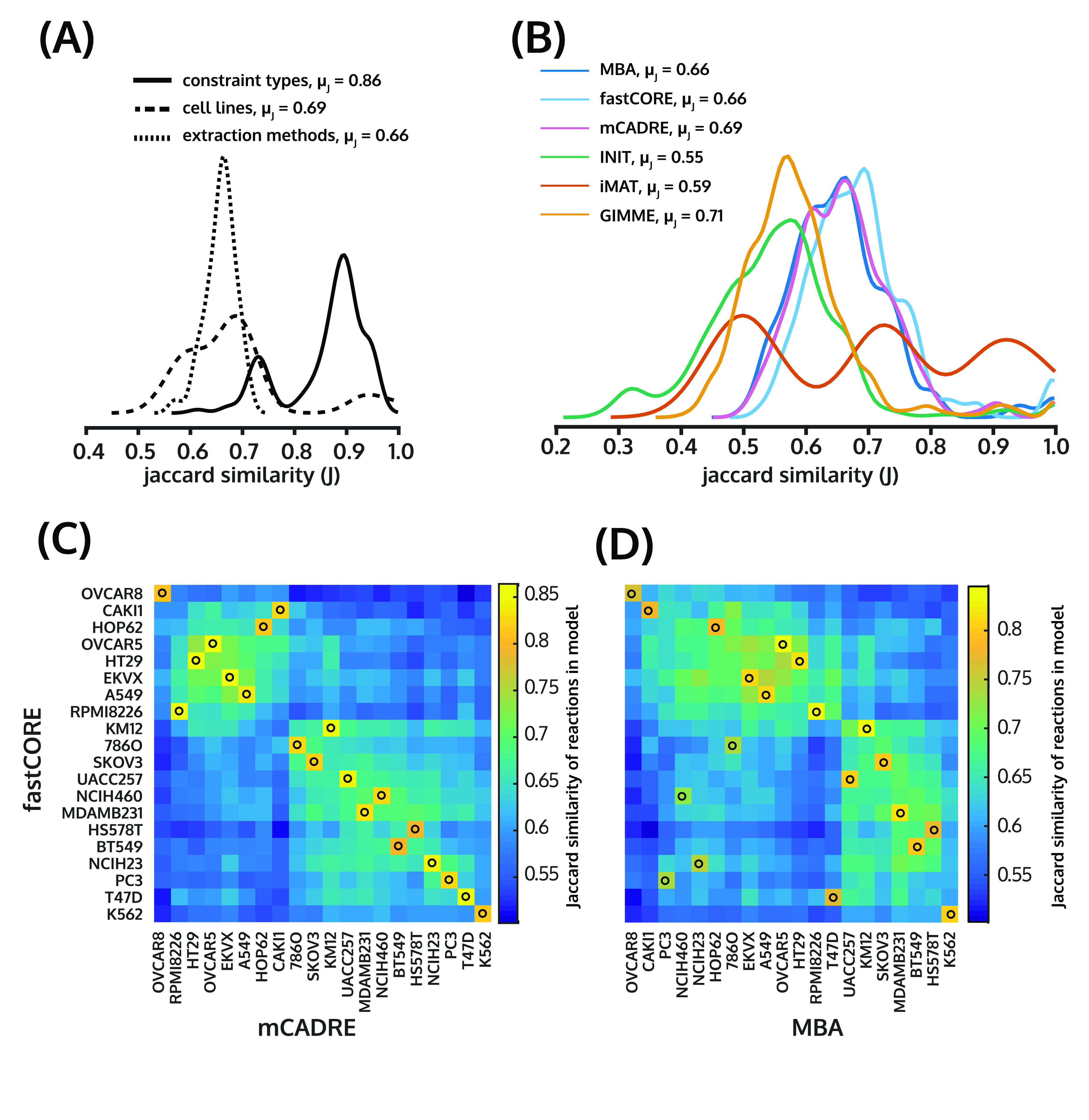

Supplement: S7 Fig — (A) Distribution of Jaccard similarity between models built using different constraint types, extraction methods, and belonging to different cell lines. (B) Distribution of Jaccard similarity between models extracted using different extraction methods. (C) Heatmap of Jaccard similarity between models extracted using mCADRE and fastCORE. (D) Heatmap of Jaccard similarity between models extracted using MBA and fastCORE. (JPG) [file pcbi.1007764.s010.jpg]

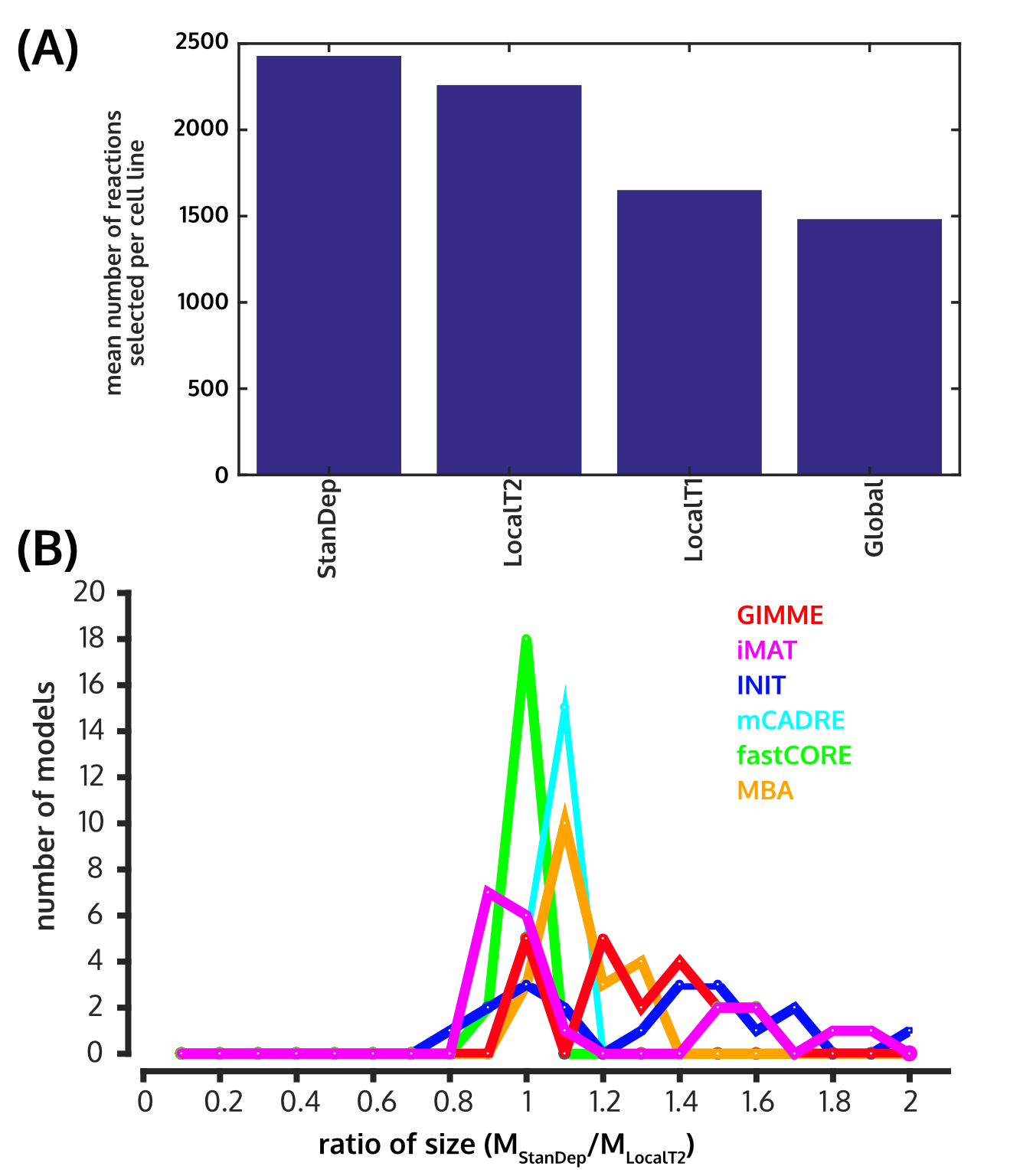

Supplement: S8 Fig — Comparison of StanDep (A) core reaction lists and (B) models with localT2. (A) On y-axis, mean number of core reactions across all 44 NCI-60 cancer cell lines. (B) The size of the models is determined by number of reactions in the model. (JPG) [file pcbi.1007764.s011.jpg]

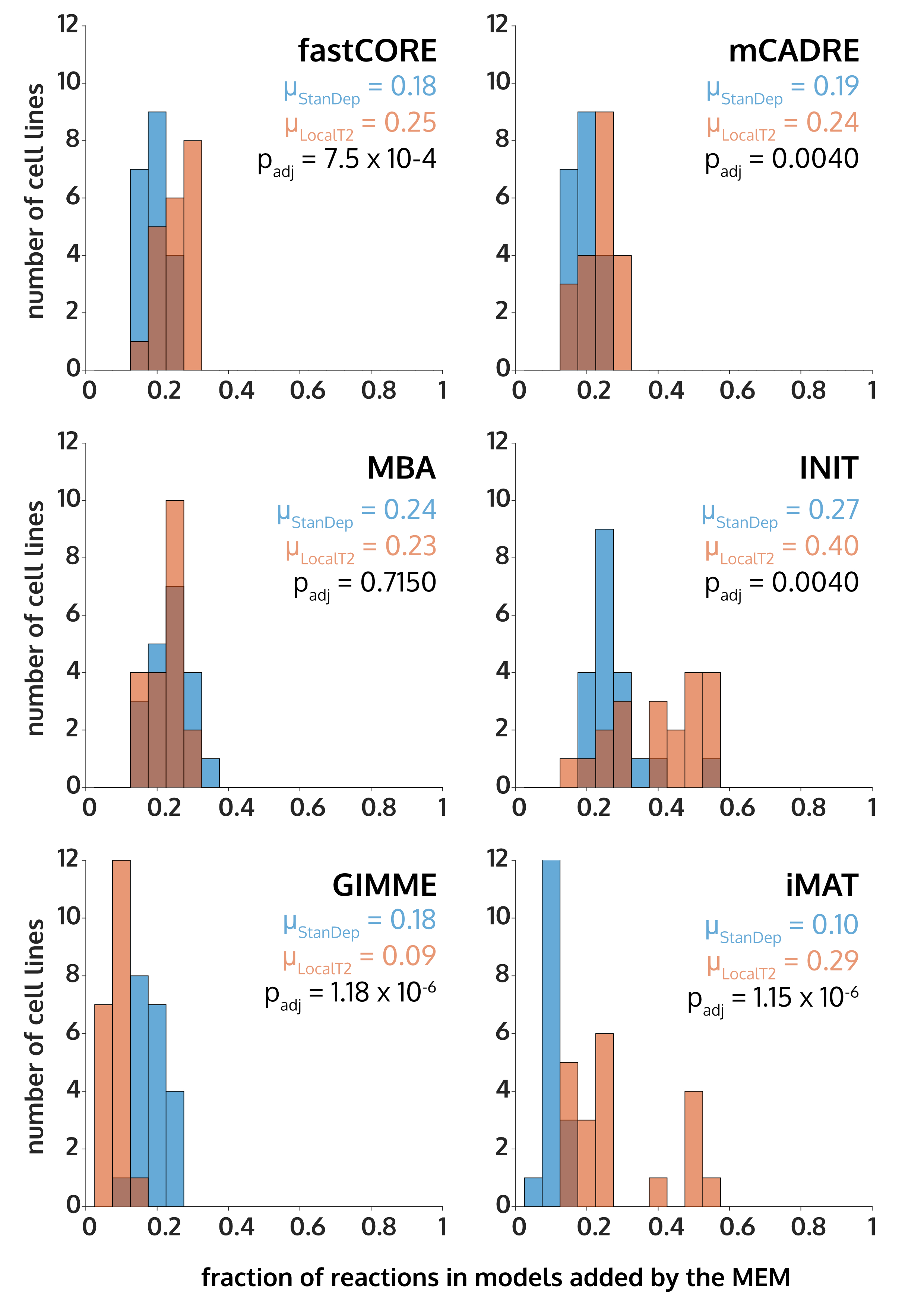

Supplement: S9 Fig — Except GIMME, the overall dissimilarity between core reaction lists and the extracted models are at least similar (MBA) or lower (fastCORE, mCADRE, INIT, and iMAT) than the same for localT2 models. (JPG) [file pcbi.1007764.s012.jpg]

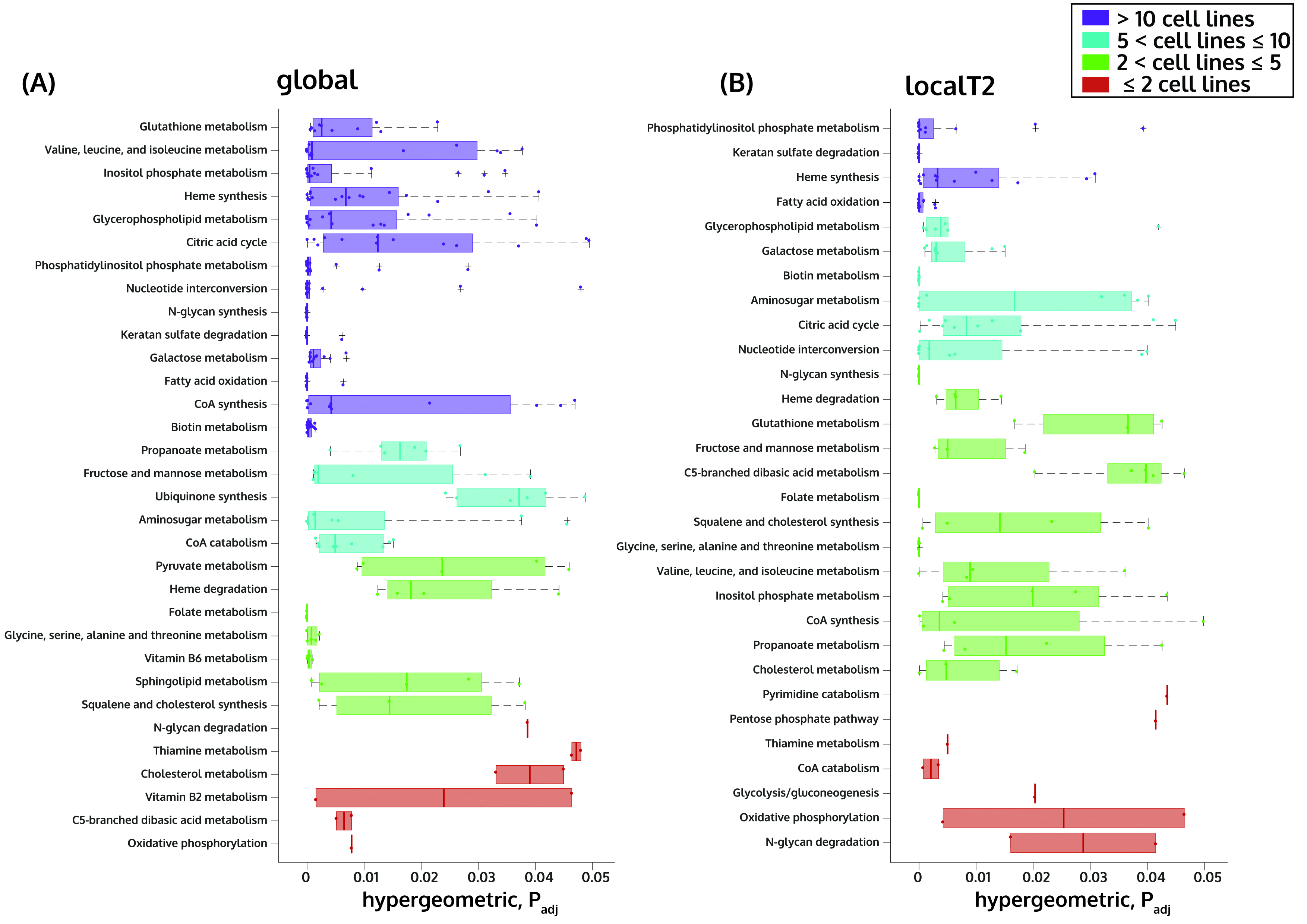

Supplement: S10 Fig — Box plot of number of cell lines in which housekeeping reactions differentially present and enriched in StanDep-derived core reaction lists compared to that of (A) global or (B) localT2. Each dot represents one cell line where the housekeeping reactions differentially present in StanDep belonging to a pathway were statistically significant. The colors indicate number of cell lines where the pathway was significant. The p-values were corrected using BHFDR. (JPG) [file pcbi.1007764.s013.jpg]

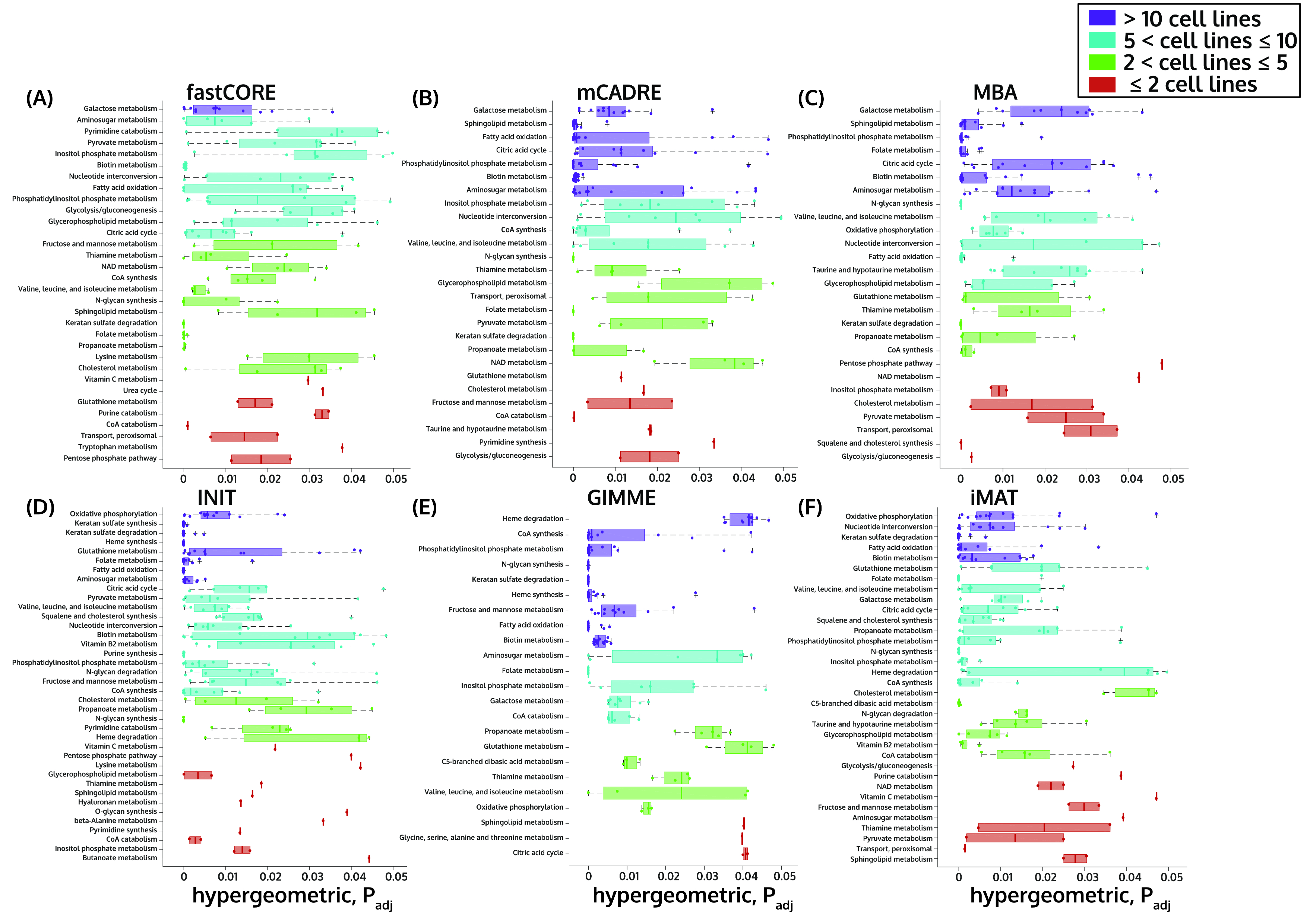

Supplement: S11 Fig — Box plot of number of cell lines in which housekeeping reactions differentially present and enriched in StanDep-derived models compared to that of localT2 thresholding using (A) fastCORE, (B) mCADRE, (C) MBA, (D) INIT, (E) GIMME, and (F) iMAT. Each dot represents one cell line where the housekeeping reactions differentially present in StanDep-derived models belonging to a pathway were statistically significant. All models were built using exometabolomic constraints. The colors indicate number of cell lines where the pathway was significant. The p-values were corrected using BHFDR. (JPG) [file pcbi.1007764.s014.jpg]

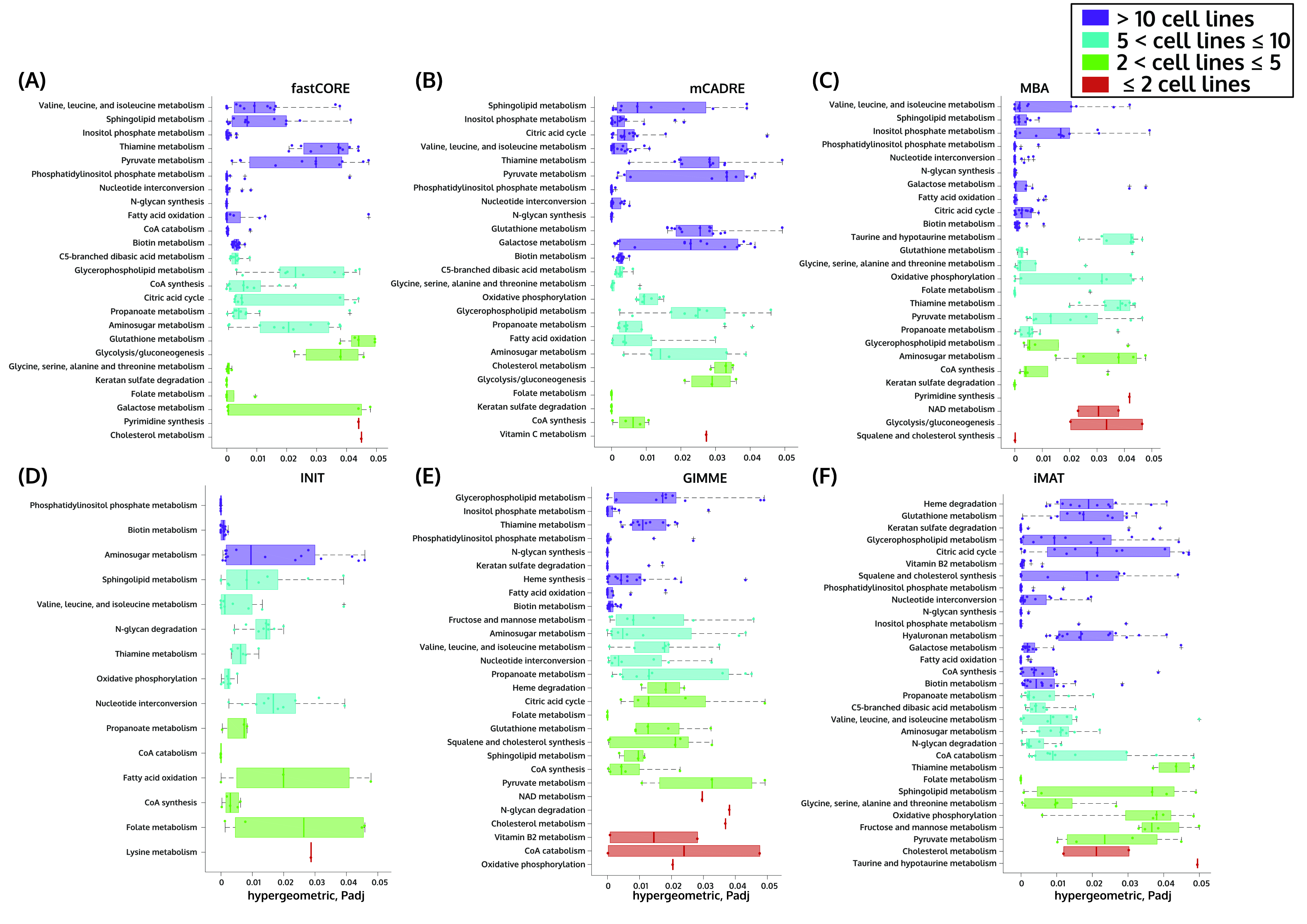

Supplement: S12 Fig — Box plot of number of cell lines in which housekeeping reactions differentially present and enriched in StanDep-derived models compared to that of global thresholding using (A) fastCORE, (B) mCADRE, (C) MBA, (D) INIT, (E) GIMME, and (F) iMAT. Each dot represents one cell line where the housekeeping reactions differentially present in StanDep-derived models belonging to a pathway were statistically significant. All models were built using exometabolomic constraints. The colors indicate number of cell lines where the pathway was significant. The p-values were corrected using BHFDR. (JPG) [file pcbi.1007764.s015.jpg]

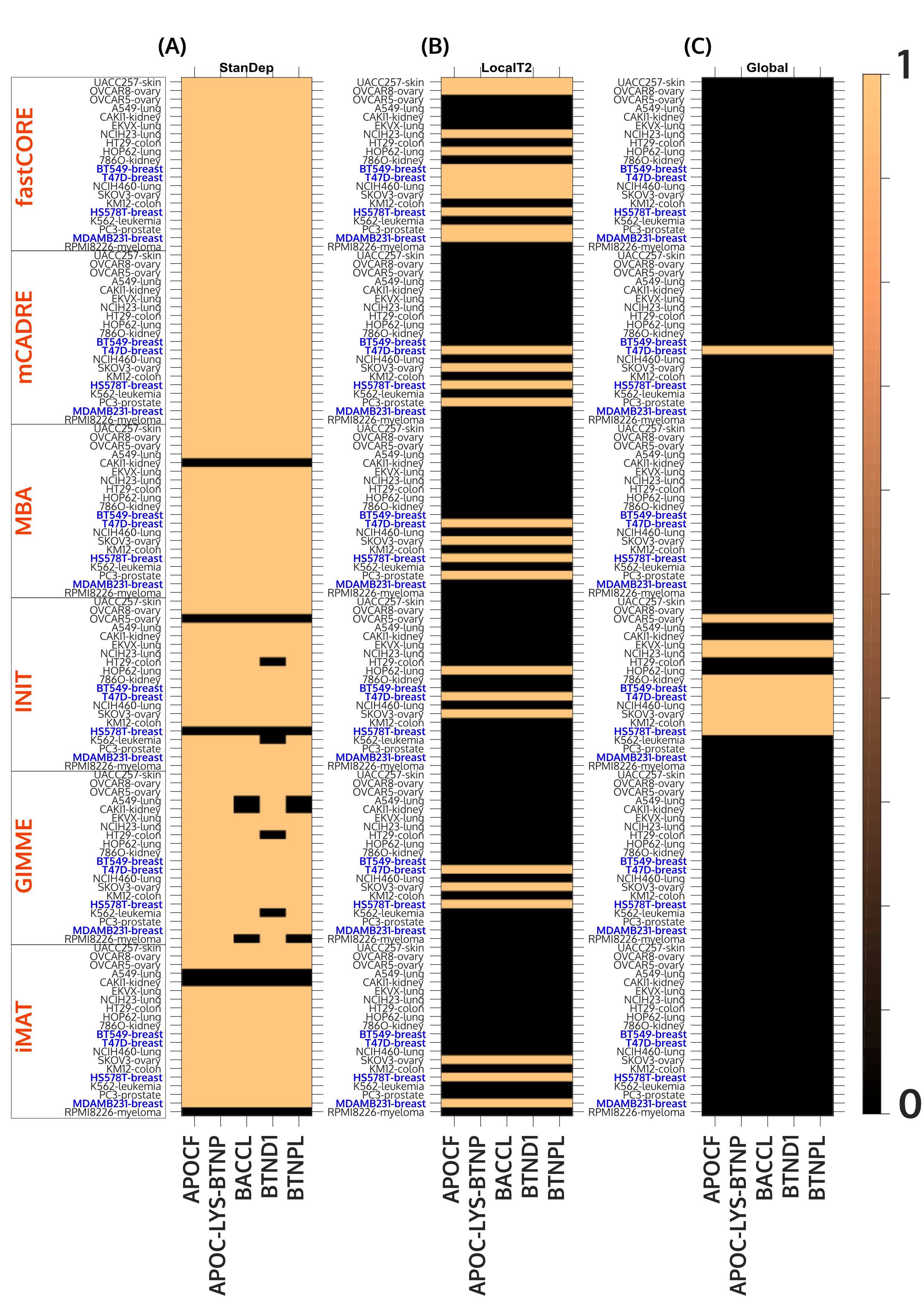

Supplement: S13 Fig — Binary heatmap of reactions from Biotin metabolism present in models built using (A) StanDep, (B) LocalT2, (C) Global and different extraction methods for each of the 20 cancer cell lines. Black indicates the absence of reaction in the model and color indicates presence. Highlighted in blue are breast cancer cell line models where BTND1 has been identified as a marker. (JPG) [file pcbi.1007764.s016.jpg]

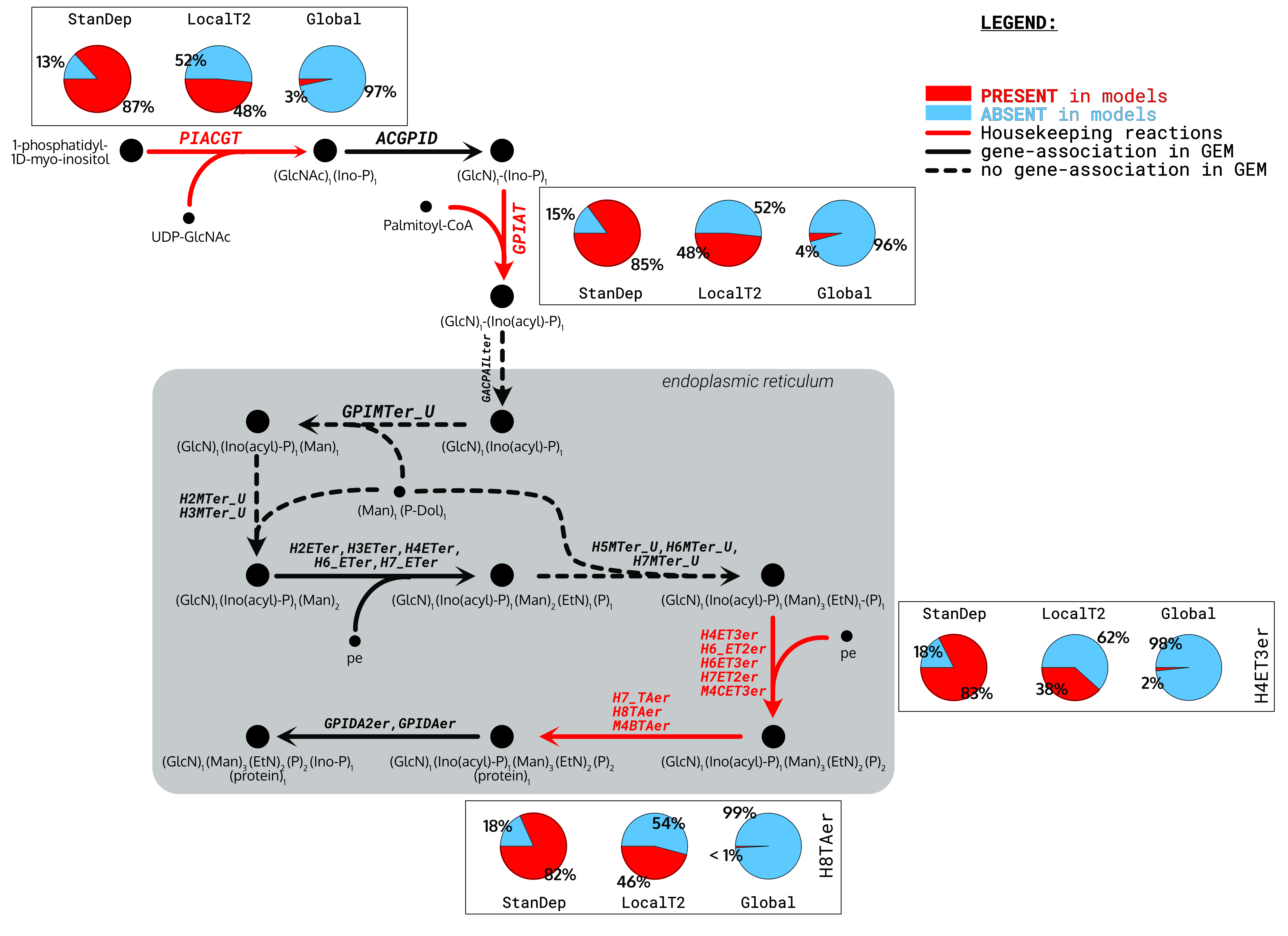

Supplement: S14 Fig — Reactions part of Glycosylphosphatidylinositol-anchor biosynthesis (phosphatidylinositol phosphate metabolism in Recon 2.2) and their coverage across models (insets containing pie plots) built using StanDep (left), localT2 (middle), and global (right). The genome-scale model (GEM) used here was Recon2.2. Housekeeping reactions are given in red, gene-associated reactions are in black, and reactions with no gene associated are given in dashed arrows. The pie plots show percentage of models (cell line-extraction method = 120 models in each pie plot) that contained (red) or did not contain (blue) the reaction. (JPG) [file pcbi.1007764.s017.jpg]

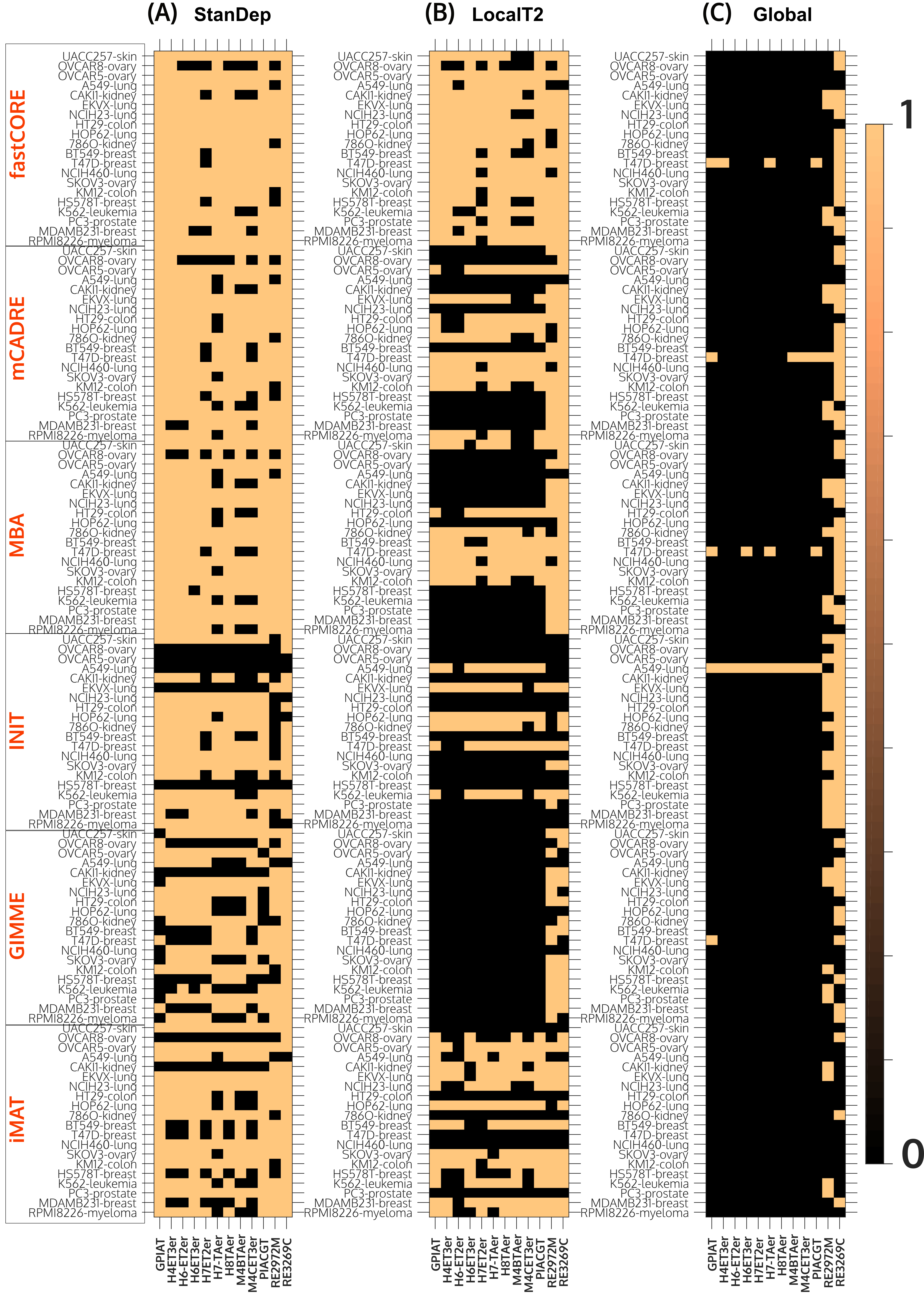

Supplement: S15 Fig — Binary heatmap of reactions from Phosphatidylinositol phosphate metabolism present in models built using (A) StanDep, (B) LocalT2, (C) Global and different extraction methods for each of the 20 cancer cell lines. Black indicates the absence of reaction in the model and color indicates presence. Highlighted in blue are breast cancer cell line models where BTND1 has been identified as a marker. (JPG) [file pcbi.1007764.s018.jpg]

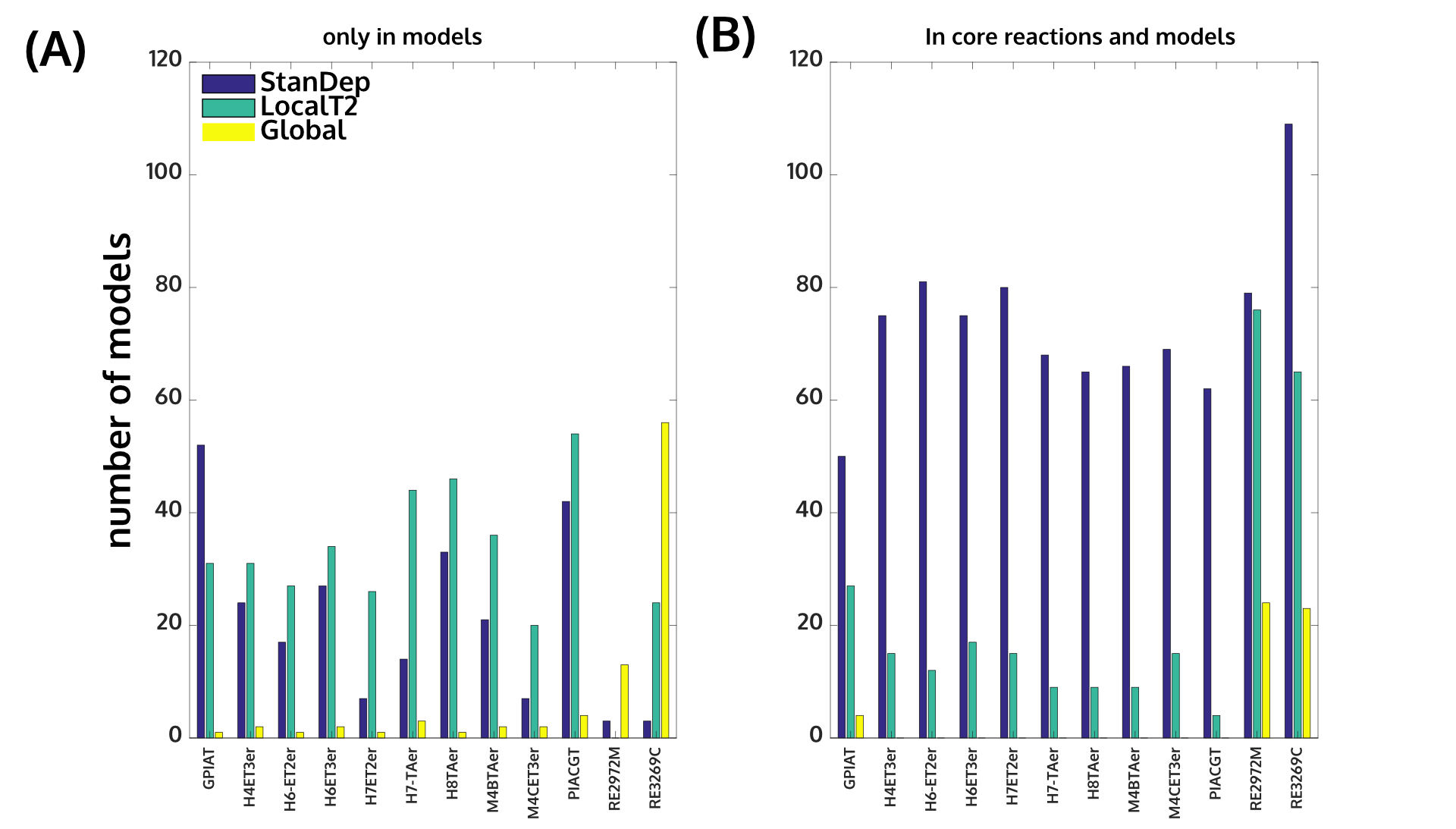

Supplement: S16 Fig — Source of the housekeeping reactions from Phosphatidylinositol phosphate metabolism) in each of the models built using StanDep (blue), localT2 (cyan) and global (yellow) as either due to extraction method (i.e. only in the model but not in core reaction list, A) or due to core reaction list (i.e. the reaction is in the model because it was present in the core reaction list, B). As seen, localT2 and global-derived models were likely to include due to extraction methods, yet the reactions were generally present in fewer models compared to those derived using StanDep. (JPG) [file pcbi.1007764.s019.jpg]

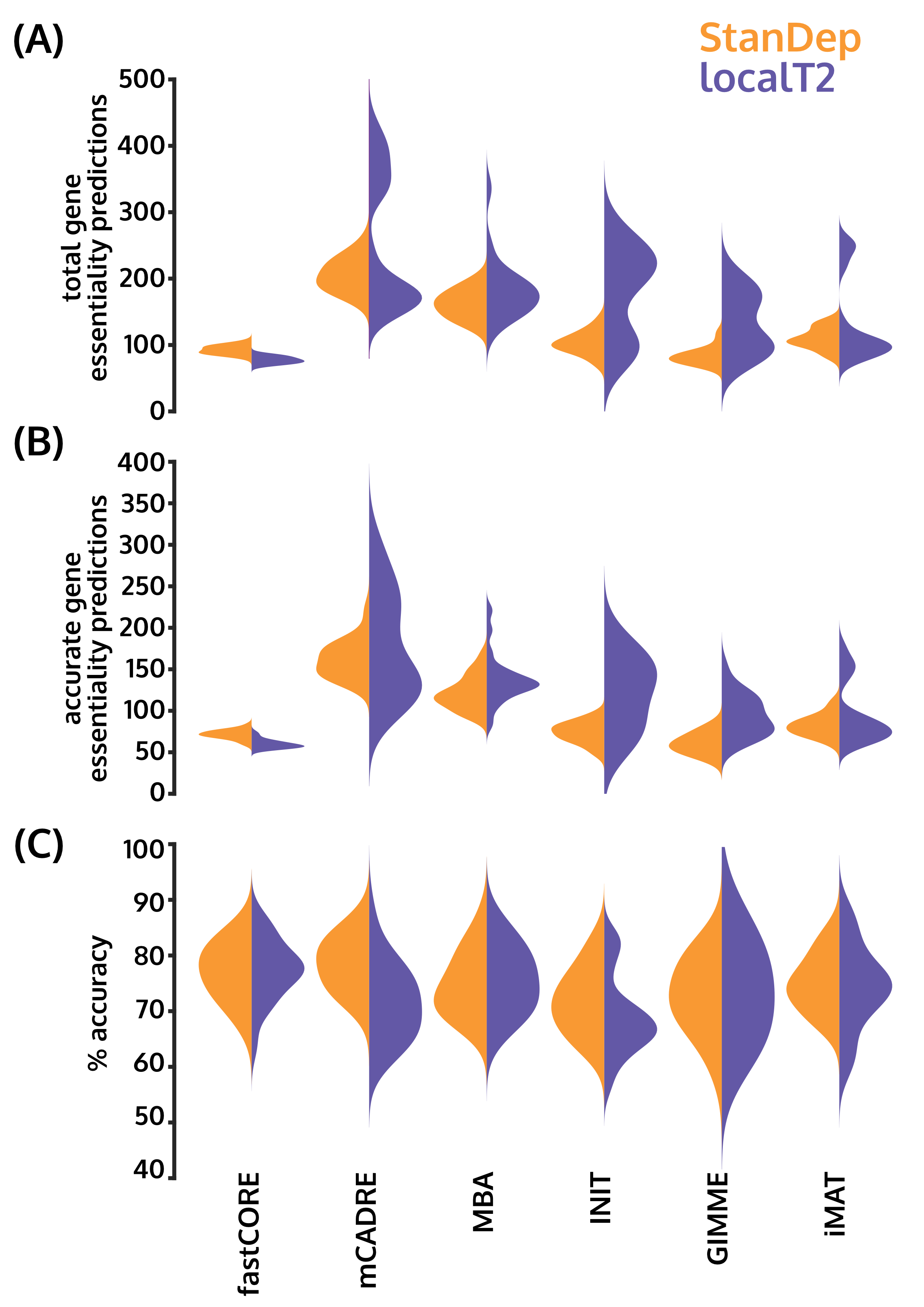

Supplement: S17 Fig — (A) Distribution of total gene essentiality predictions generated by models. (A) Distributions of accurate gene essentiality predictions generated by models. (C) Distribution of accuracy of gene essentiality predictions. The violin plots represent distributions of StanDep (orange) and localT2 (purple) models. (JPG) [file pcbi.1007764.s020.jpg]

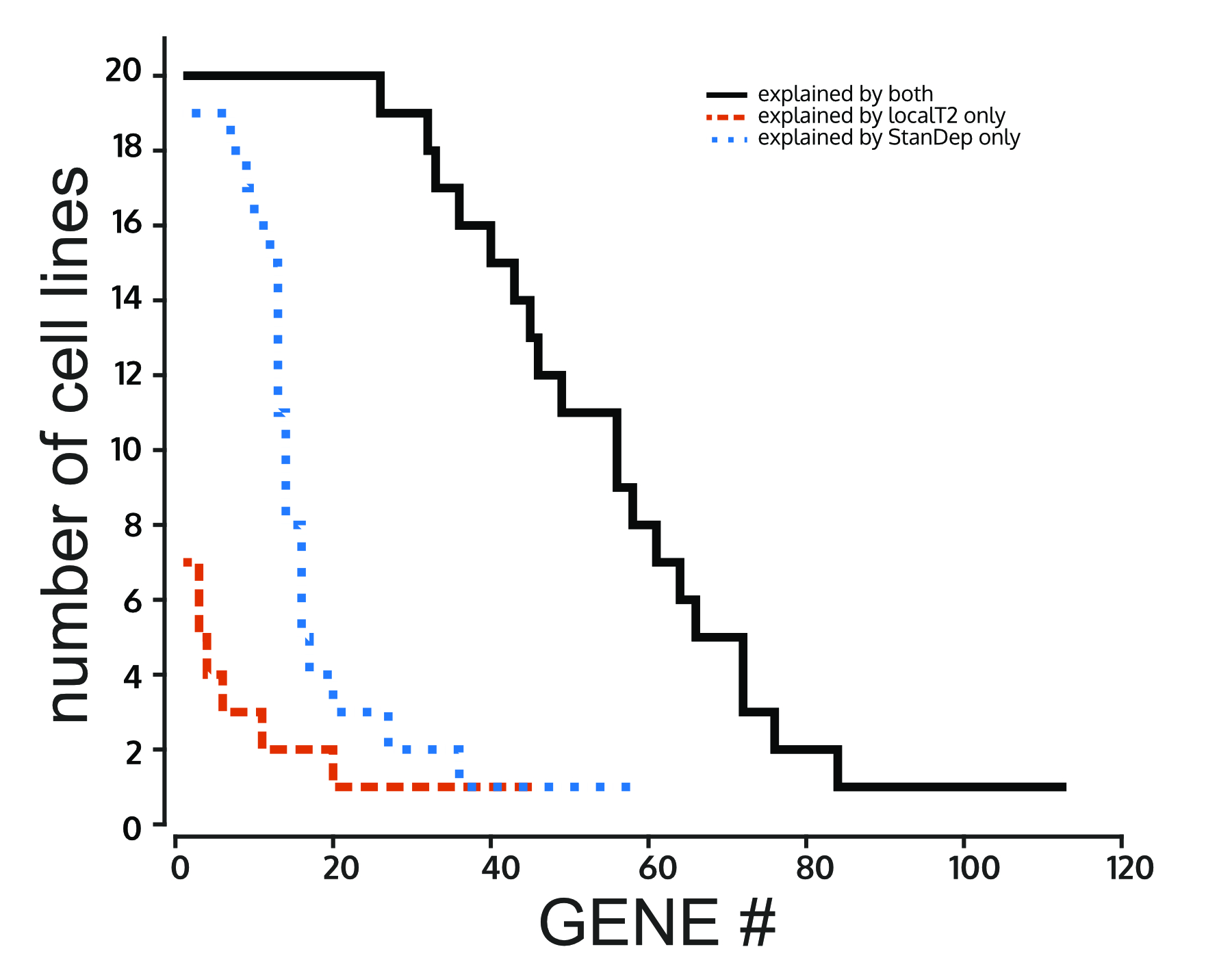

Supplement: S18 Fig — The figure only contains fastCORE models. (JPG) [file pcbi.1007764.s021.jpg]

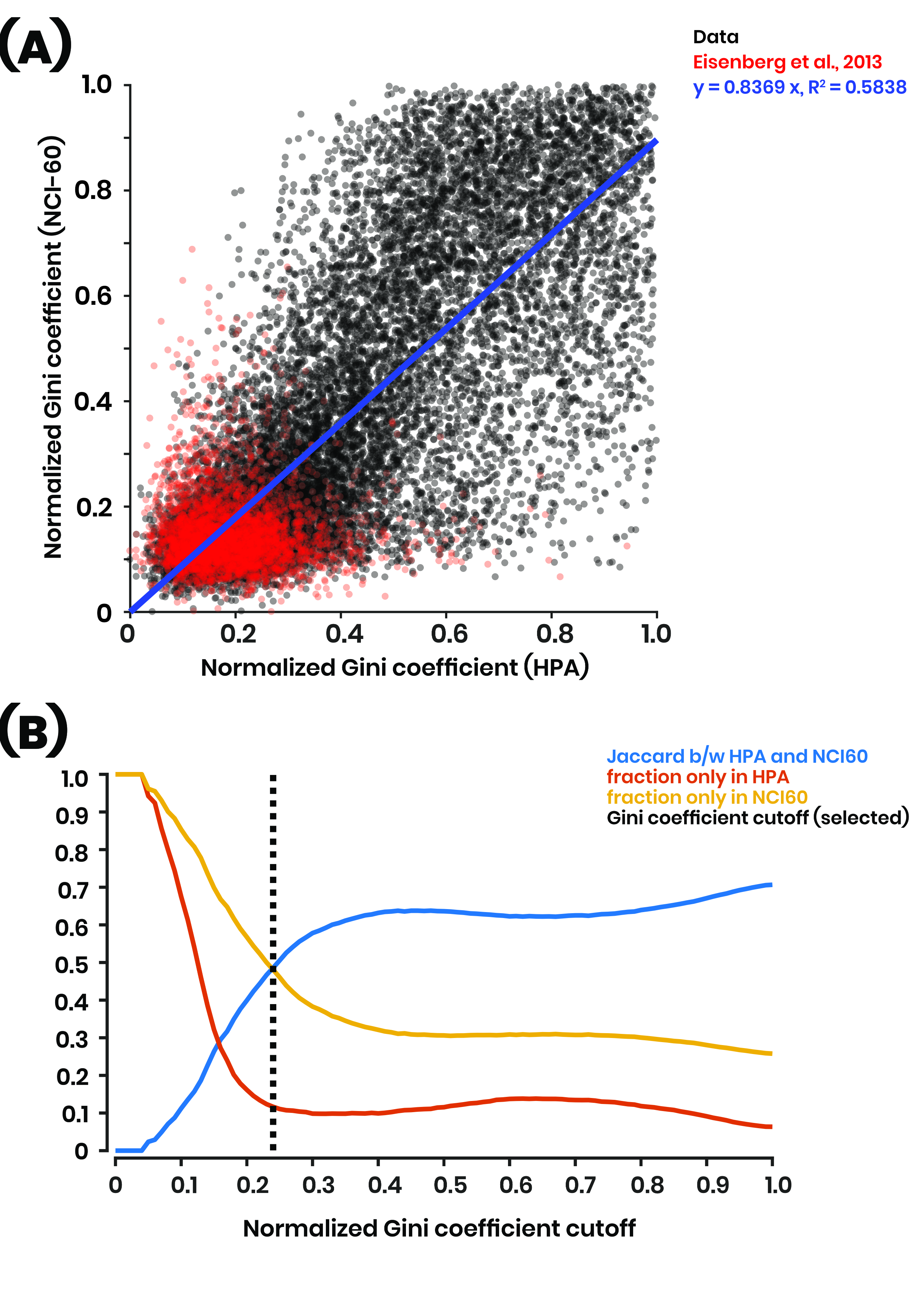

Supplement: S19 Fig — Identifying a Gini coefficient (GC) cutoff from two different datasets. (A) Normalized GC of housekeeping genes listed by Eisenberg & Levanon [22] when using HPA or Klijn et al. (NCI-60) transcriptomic data. Most of these genes have low GC value. (B) GC cutoff at the point where the Jaccard similarity in the housekeeping genes in both datasets intersected the highest fraction of novel housekeeping genes identified by either dataset. This value was 0.24. (JPG) [file pcbi.1007764.s022.jpg]

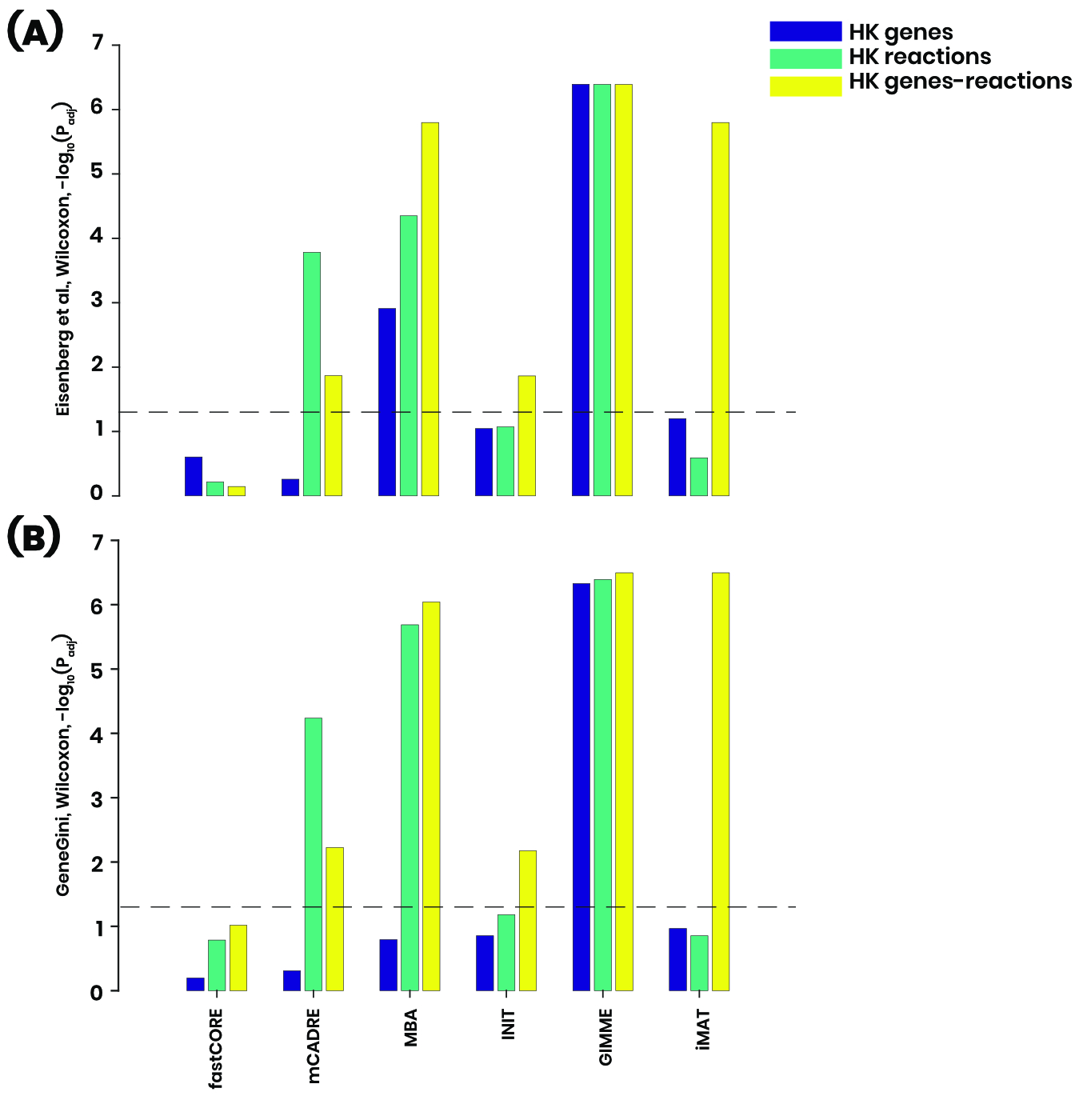

Supplement: S21 Fig — Comparison of coverage of (A) Eiseberg- and (B)GINI-housekeeping reactions in StanDep vs localT2 models from different extraction methods. The source of housekeeping reactions does not affect the statistical significance of the model comparisons. (JPG) [file pcbi.1007764.s024.jpg]

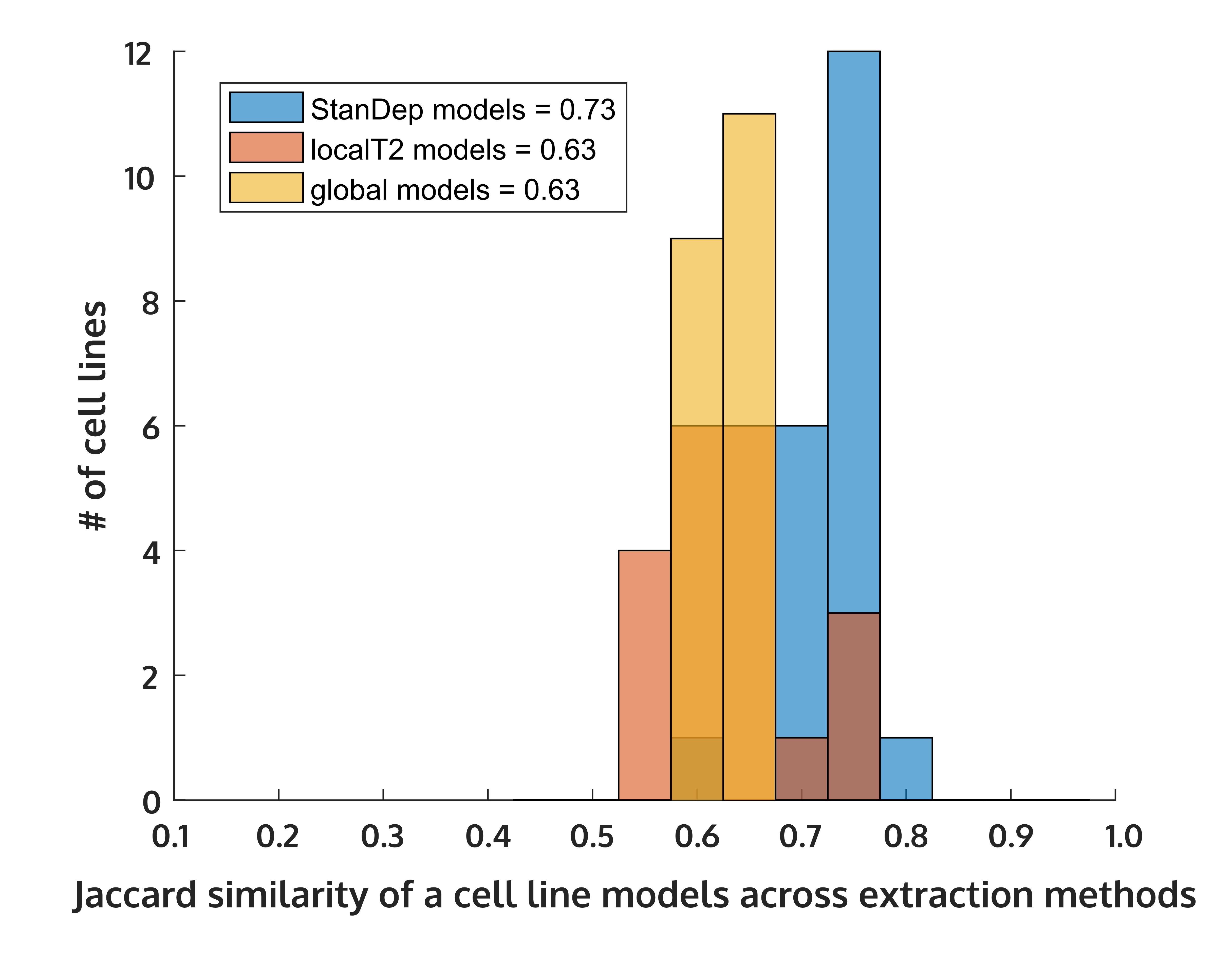

Supplement: S22 Fig — The figure shows higher consensus among models built using StanDep but different extraction methods. (JPG) [file pcbi.1007764.s025.jpg]

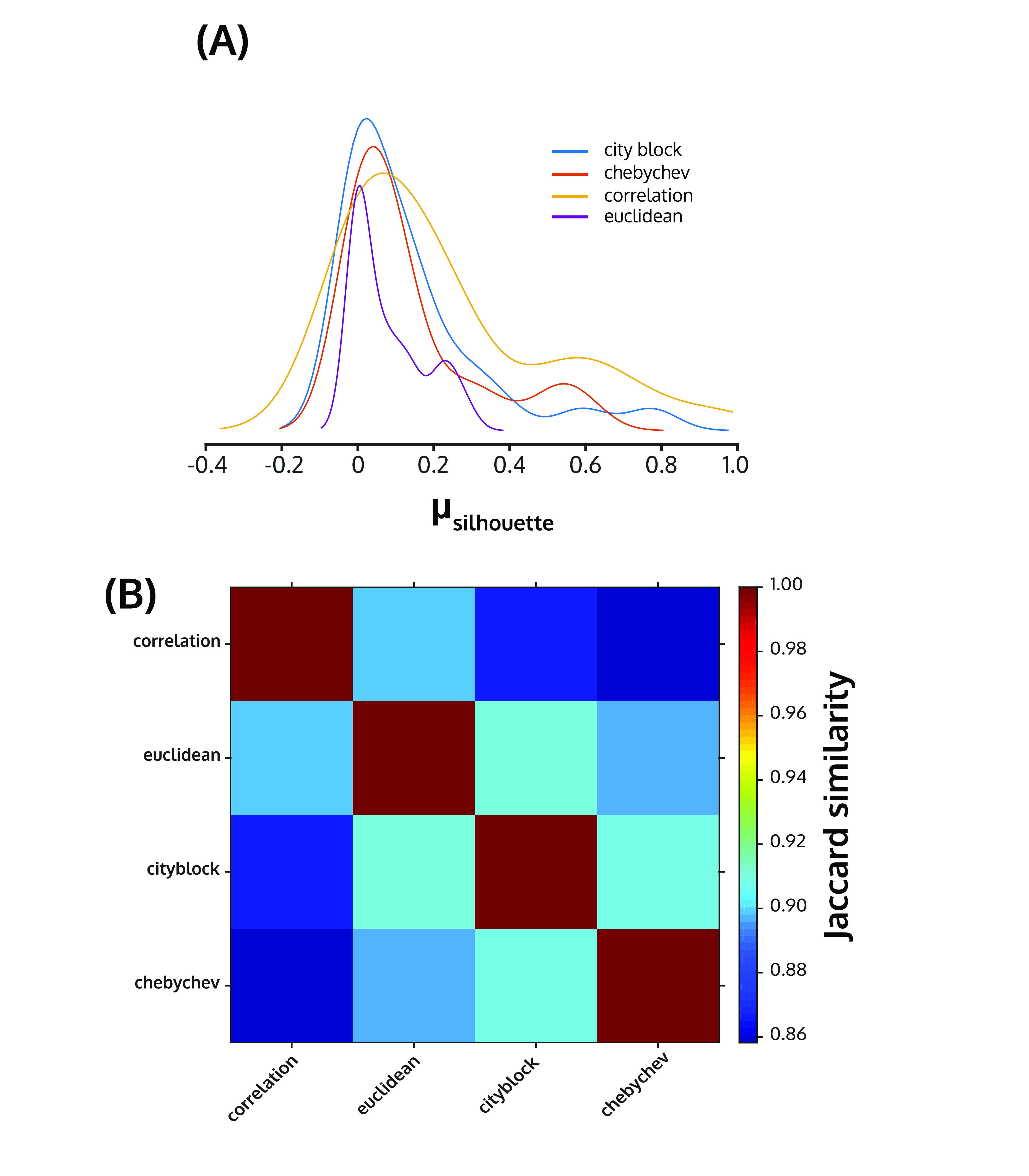

Supplement: S23 Fig — Comparison of Jaccard similarity for core reaction list of 44 cancer cell lines between (B) various distance metrics and (A) silhouette value for quality of clusters for each distance metric when using StanDep. The complete linkage method and 26 clusters were used. All distance metrics lead to over 90% mean Jaccard similarity. (JPG) [file pcbi.1007764.s026.jpg]

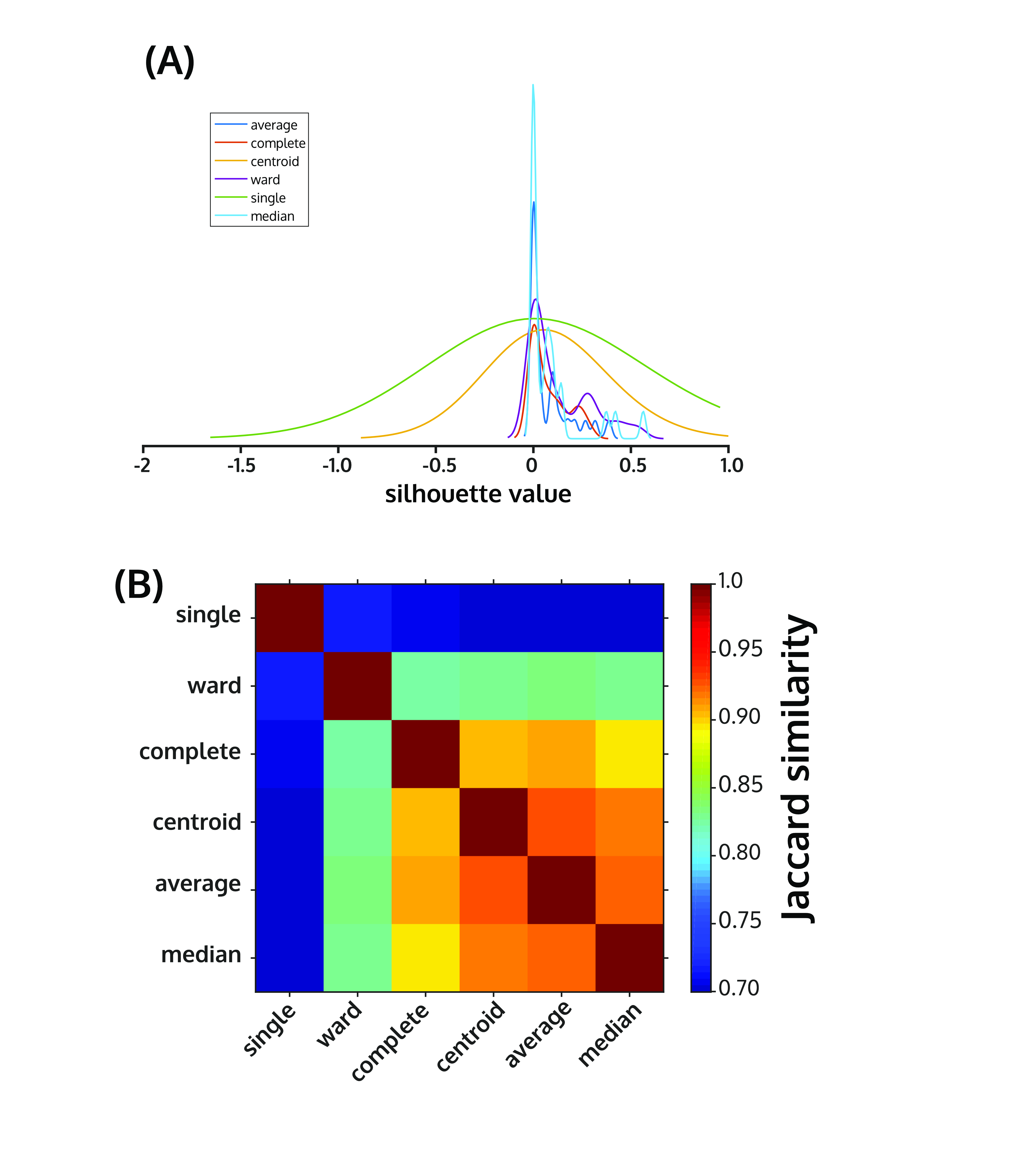

Supplement: S24 Fig — Comparison of Jaccard similarity for core reaction list of 44 cancer cell lines using (B) different linkage methods and (A) silhouette value for quality of clusters when using StanDep. The Euclidean distance method and 26 clusters were used. Complete, centroid, average, and median lead to nearly 90% mean Jaccard similarity. (JPG) [file pcbi.1007764.s027.jpg]

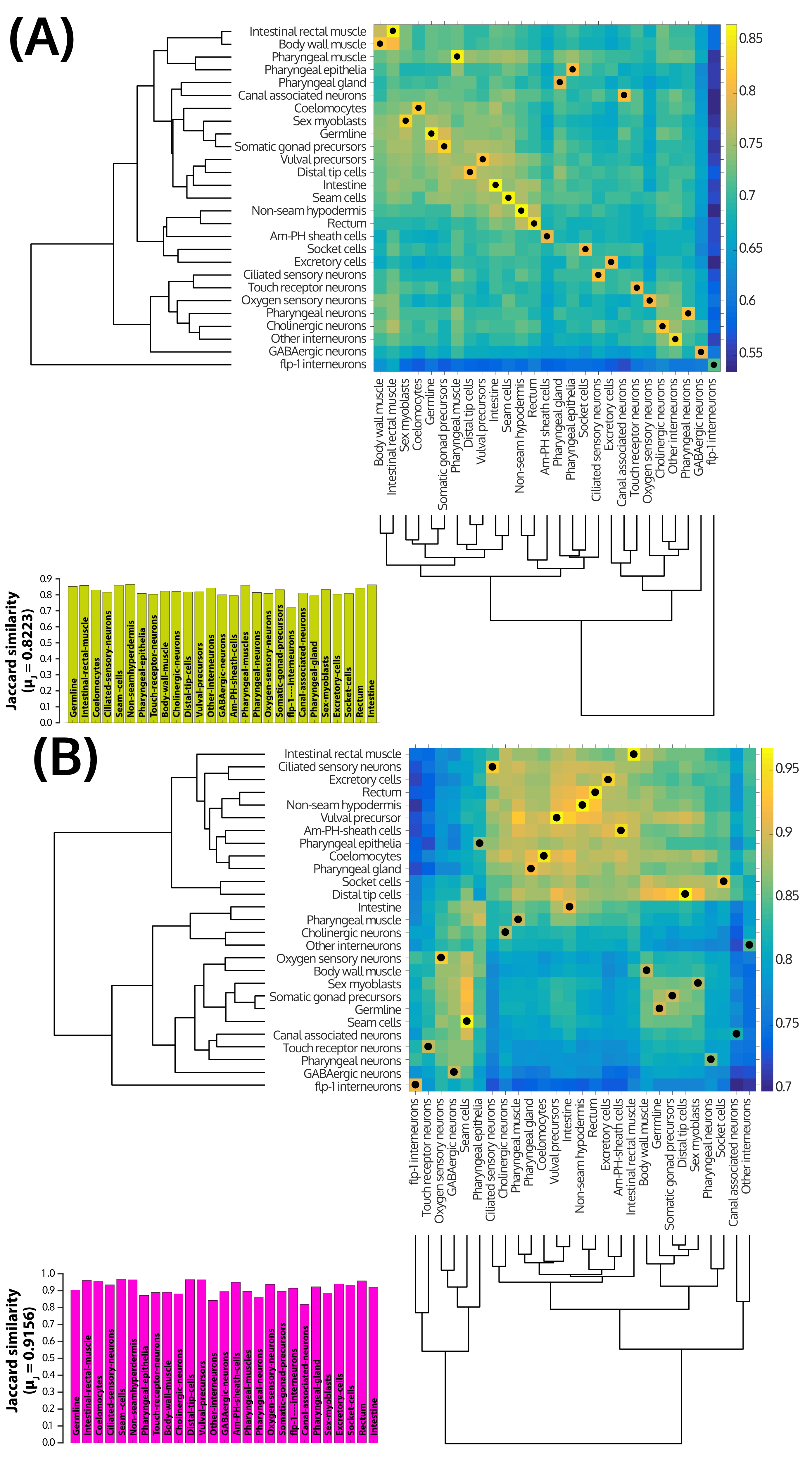

Supplement: S25 Fig — Comparison of (A) reaction content and (B) gene content of models extracted using mCADRE (x-axis) and fastCORE (y-axis). The lower inset shows the Jaccard similarity of (A) reaction or (B) gene content between these two extraction methods for each cell type. The dendrograms on left (fastCORE) and bottom (mCADRE) of the heatmaps were created using hierarchical clustering of the Jaccard similarity between content of models. The black dots on the heatmap track similar cell types across the two MEMs. The reaction content of models belonging to neuronal cell types clustered together when either MEMs were used. Models of a given cell type are best matched (black dots) with themselves across the models extracted using these two methods. (JPG) [file pcbi.1007764.s028.jpg]

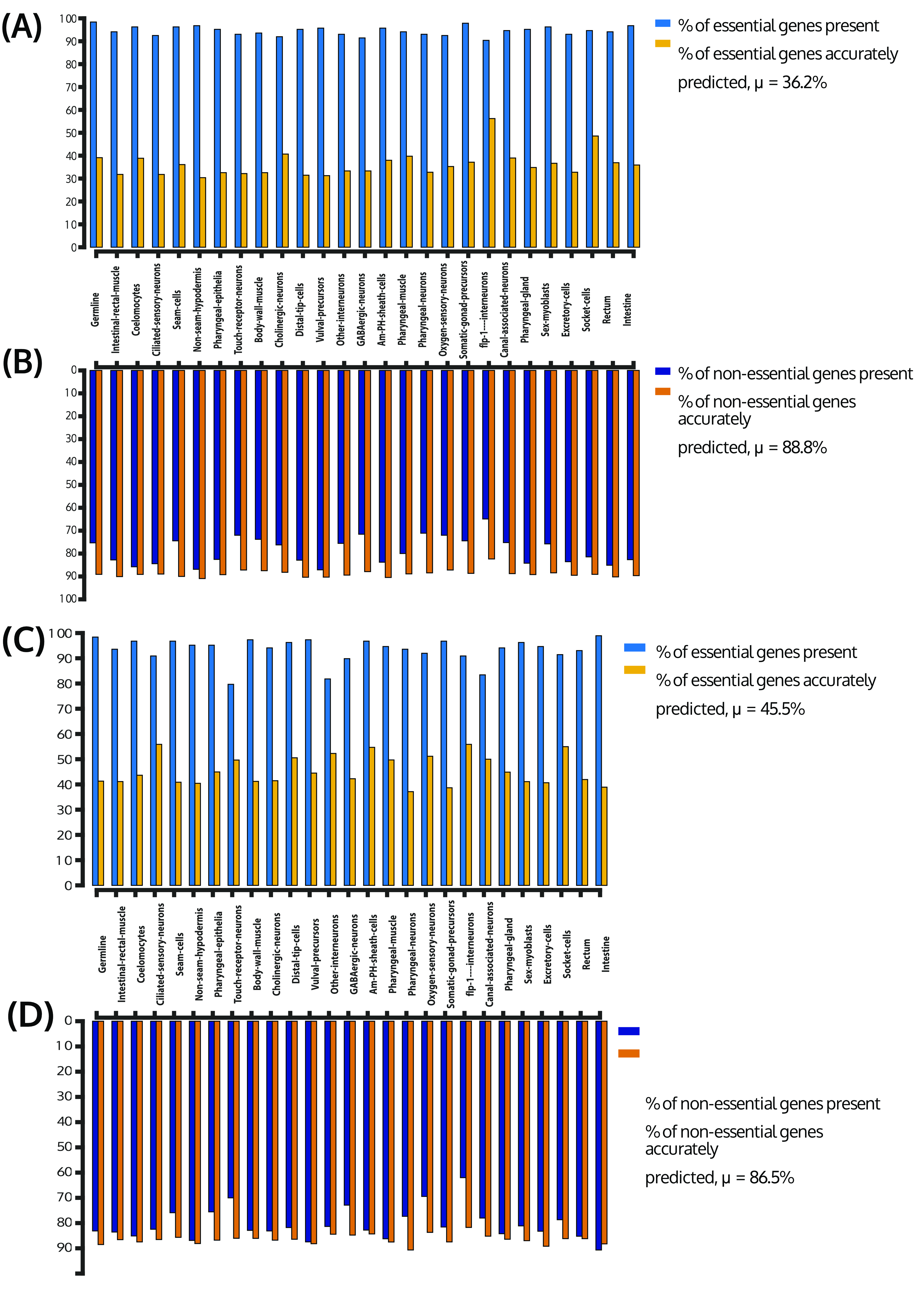

Supplement: S26 Fig — Validation of models extracted with fastCORE using (A) essential genes, (B) non-essential genes; with mCADRE using (C) essential genes, and (D) non-essential genes obtained from RNAi screens of Kamath et al. Accuracy of essential gene predictions is comparable to that of unconstrained models of NCI-60 cancer cell lines. Comparison with randomly permuted gene labels for presence of essential genes in the cell type models is presented in S29 Fig. Large fraction of essential genes predicted are present in all cell types. (JPG) [file pcbi.1007764.s029.jpg]

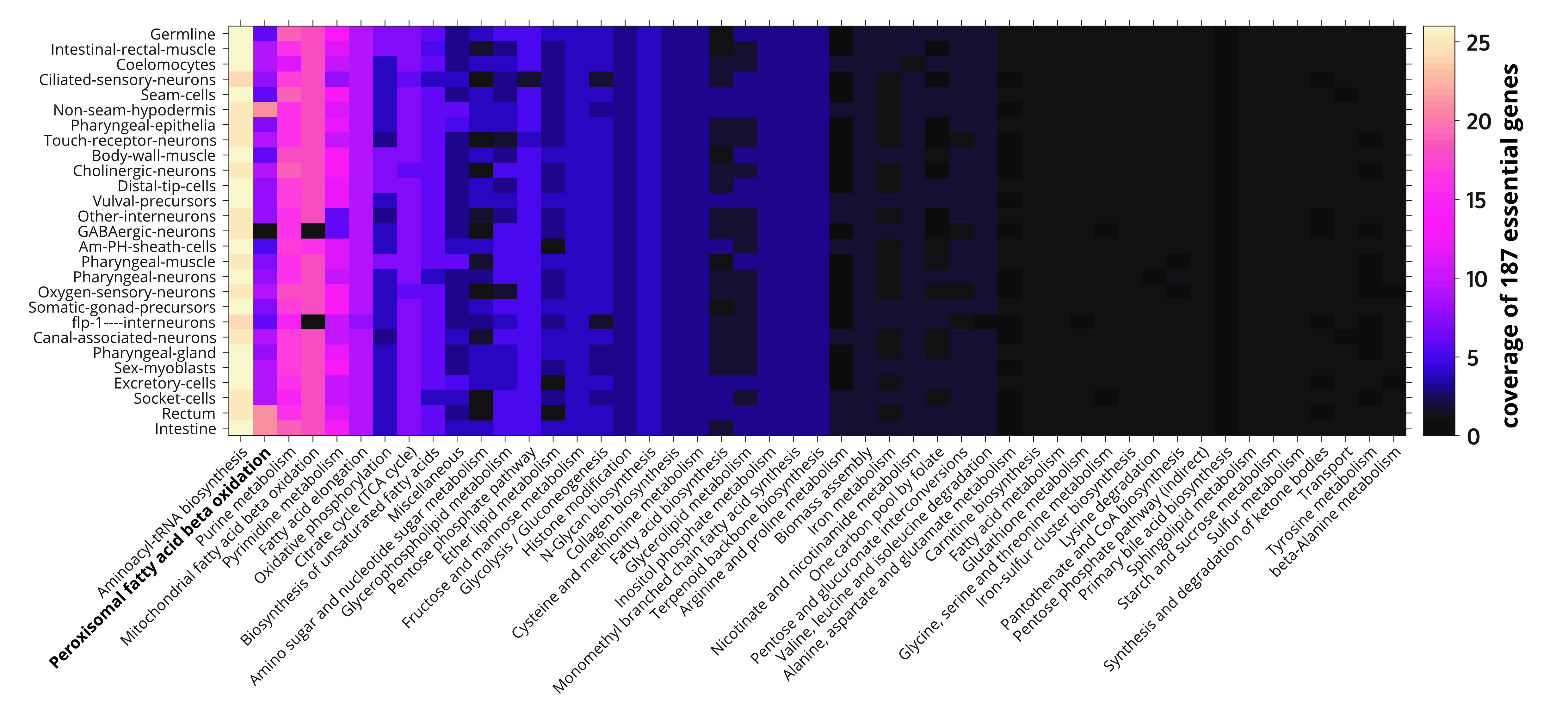

Supplement: S27 Fig — (JPG) [file pcbi.1007764.s030.jpg]

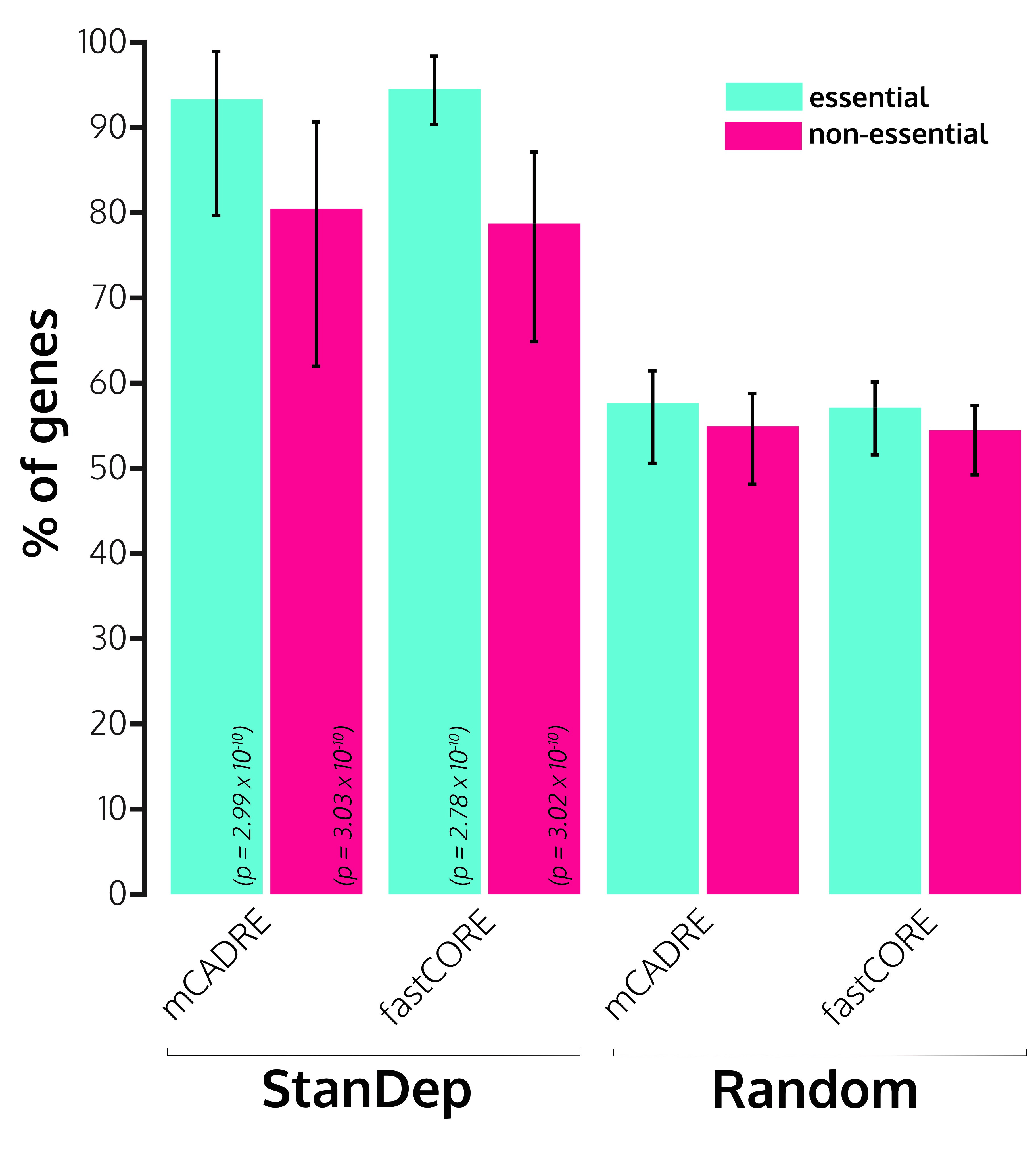

Supplement: S28 Fig — The bars represent mean percentage of 187 essential genes or 900 non-essential genes across all cell types. Error bars represent variation of the same among all cell type models. Percentage of essential and non-essential genes for models extracted using fastCORE and mCADRE is significantly different from that if models were created randomly. (JPG) [file pcbi.1007764.s031.jpg]

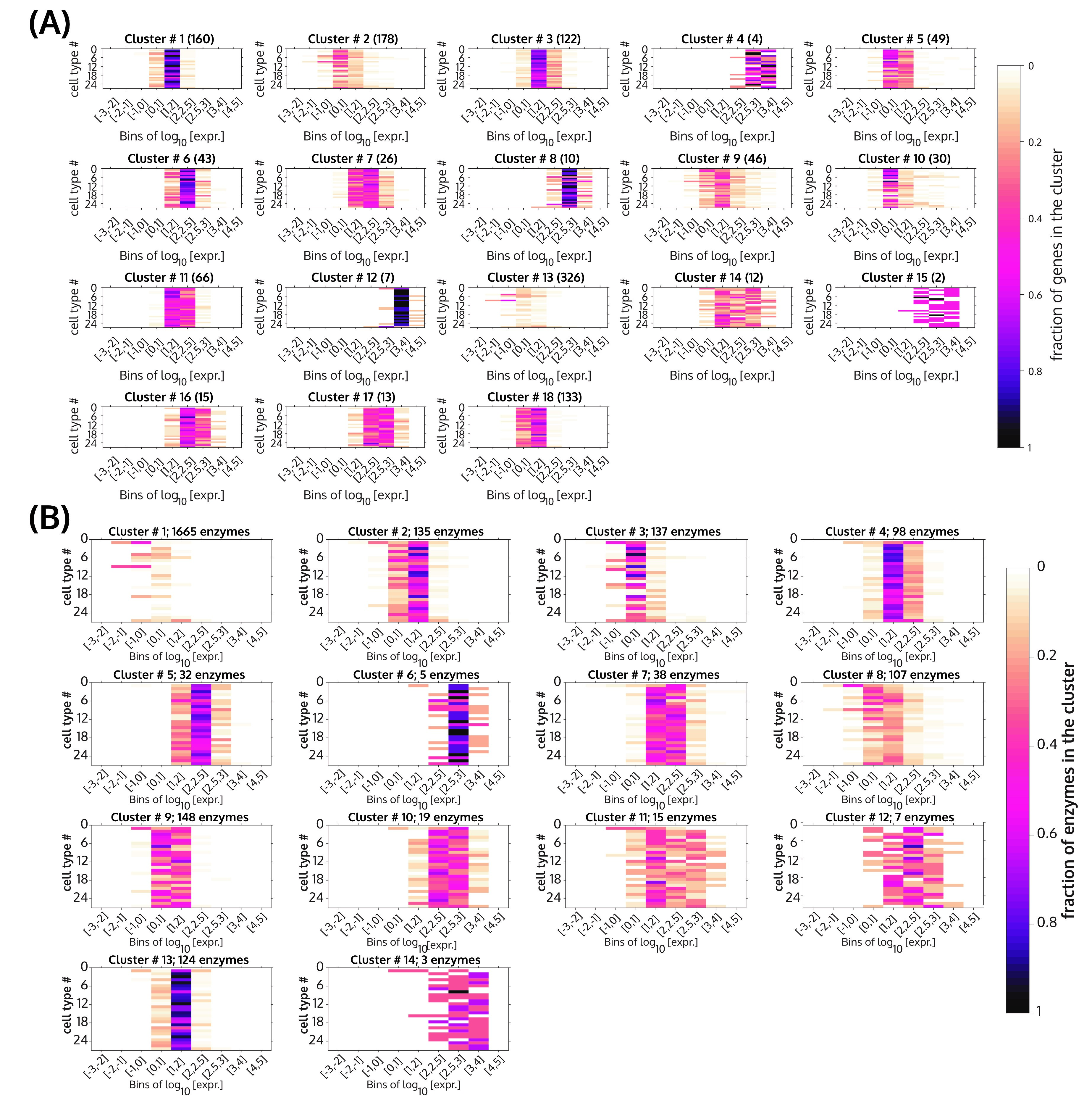

Supplement: S29 Fig — Heatmap of fraction of (A) genes or (B) enzymes in a given cluster for a given cell line (y-axis) are binned according to their log10 expression value (x-axis) for Cao et al. dataset for C. elegans. Heatmaps are shown for all (A) 18 clusters for gene expression and (B) 14 clusters for gene expression. Black represents all the genes in that cluster are binned in a certain expression range for a given cell type. White represents none of the genes in that cluster binned in a certain expression range for a given cell type. (JPG) [file pcbi.1007764.s032.jpg]

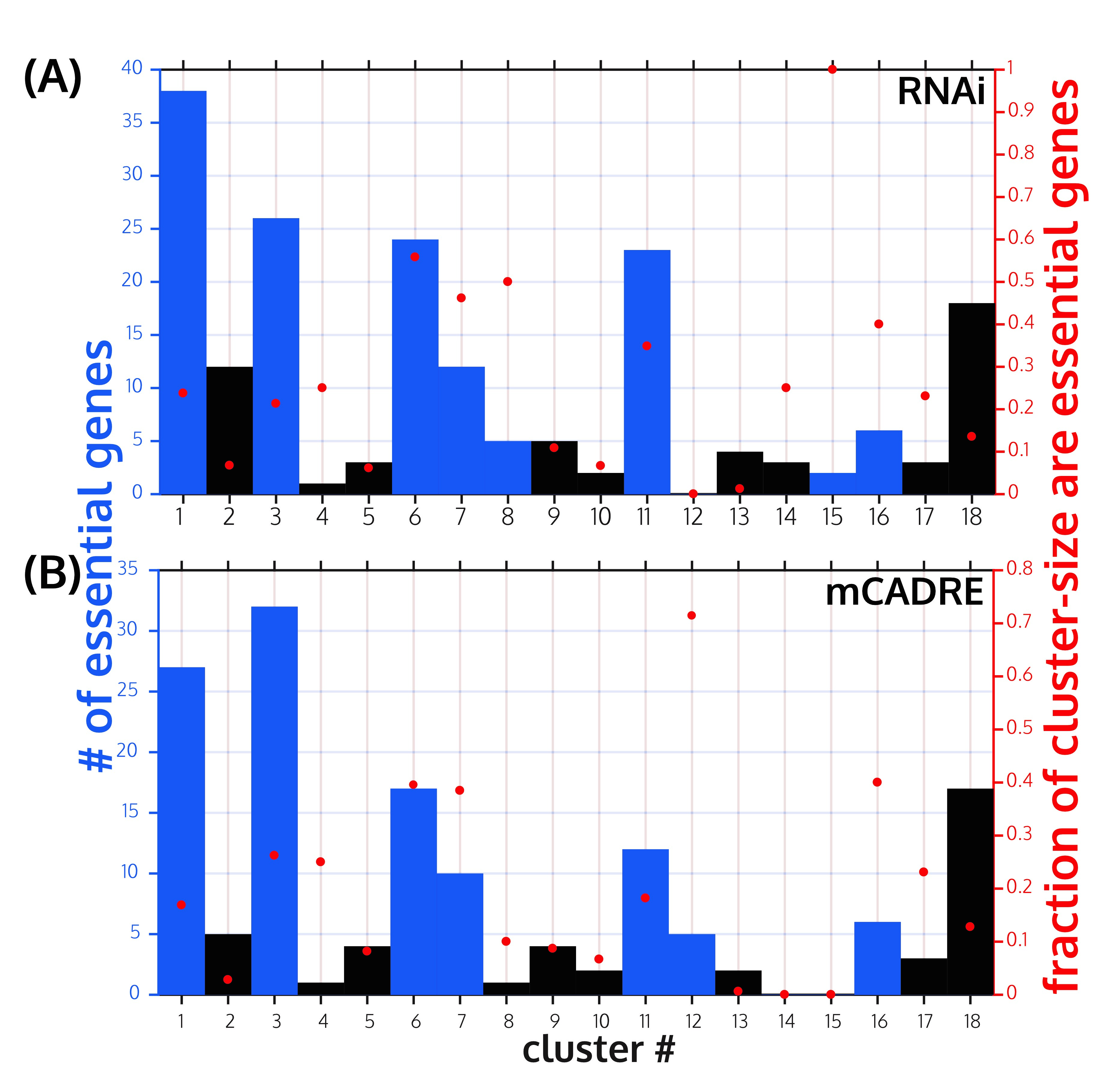

Supplement: S30 Fig — Enrichment analysis for presence of 187 whole animal essential genes (A) extracted from RNAi screens of Kamath et al., and (B) determined from models extracted using mCADRE for clusters calculated from Cao et al. C. elegans cell type sciRNA-seq data. Both analyses show enrichment in same clusters, except clusters 8 and 15 which total to only 7 genes. Clusters not enriched for whole animal essential genes are shown by black bars, while clusters enriched in whole animal essential genes are shown in blue bars. The red dots show the fraction of number of genes in each cluster are housekeeping genes (right y-axis). Enrichment was calculated as hypergeometric p-value ≤ 0.05. (JPG) [file pcbi.1007764.s033.jpg]

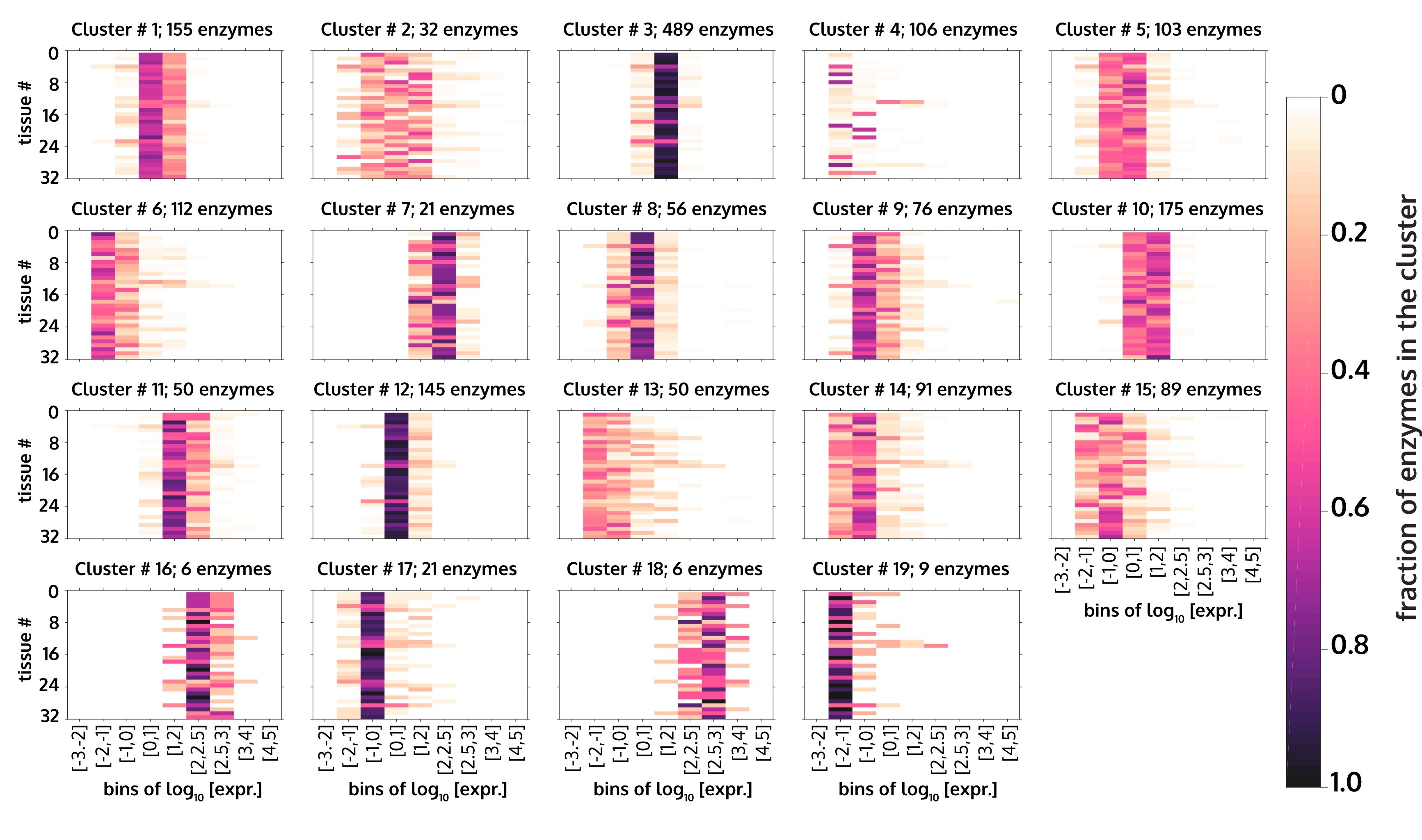

Supplement: S31 Fig — Heatmaps are shown for all 19 clusters. Black represents all the enzymes in that cluster are binned in a certain expression range for a given cell line. White represents none of the enzymes in that cluster binned in a certain expression range for a given tissue. (JPG) [file pcbi.1007764.s034.jpg]

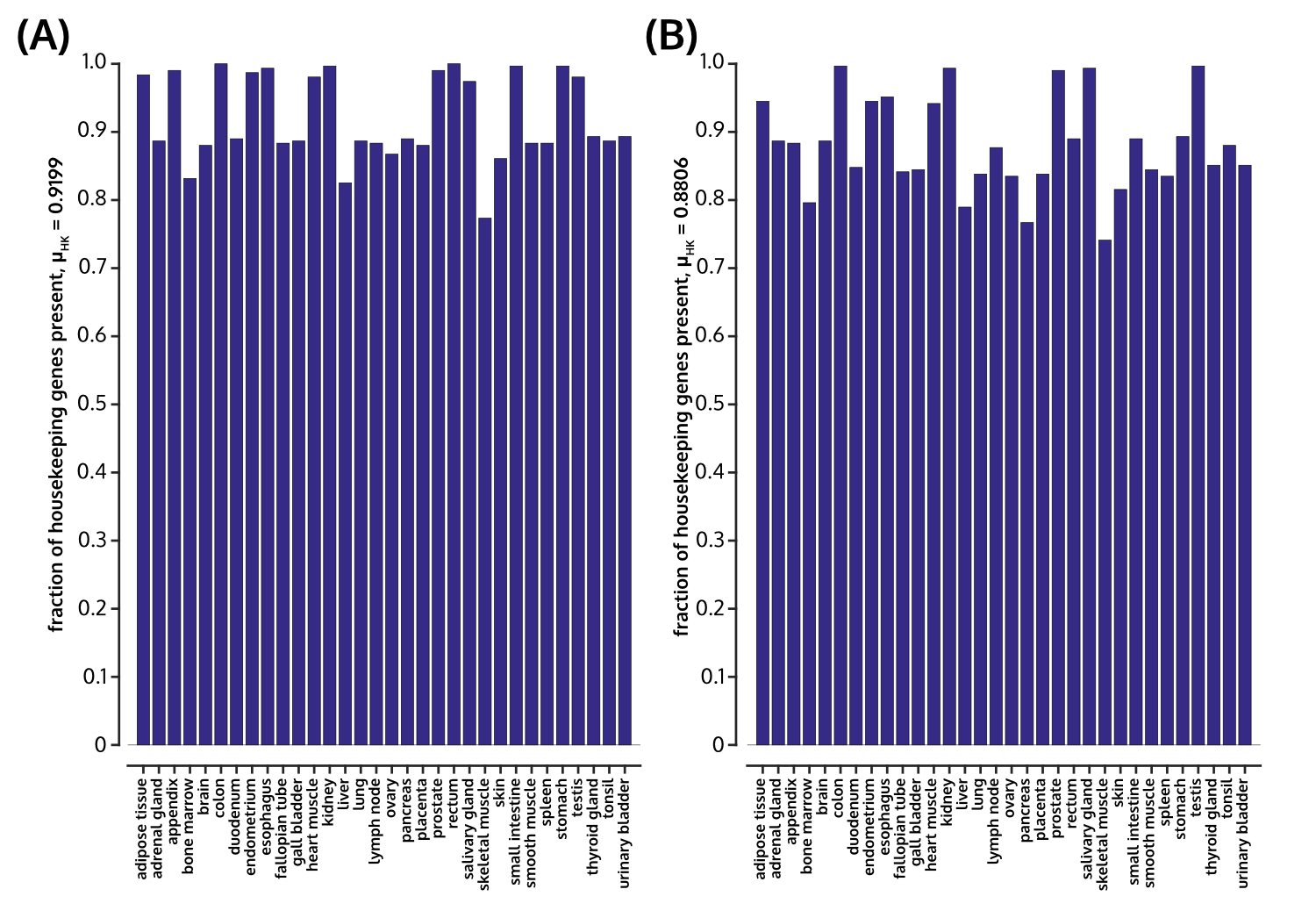

Supplement: S32 Fig — StanDep models of human tissues (transcriptomic data: HPA [6]) extracted using (A) fastCORE and (B) mCADRE contain over 88% of housekeeping reactions. (JPG) [file pcbi.1007764.s035.jpg]

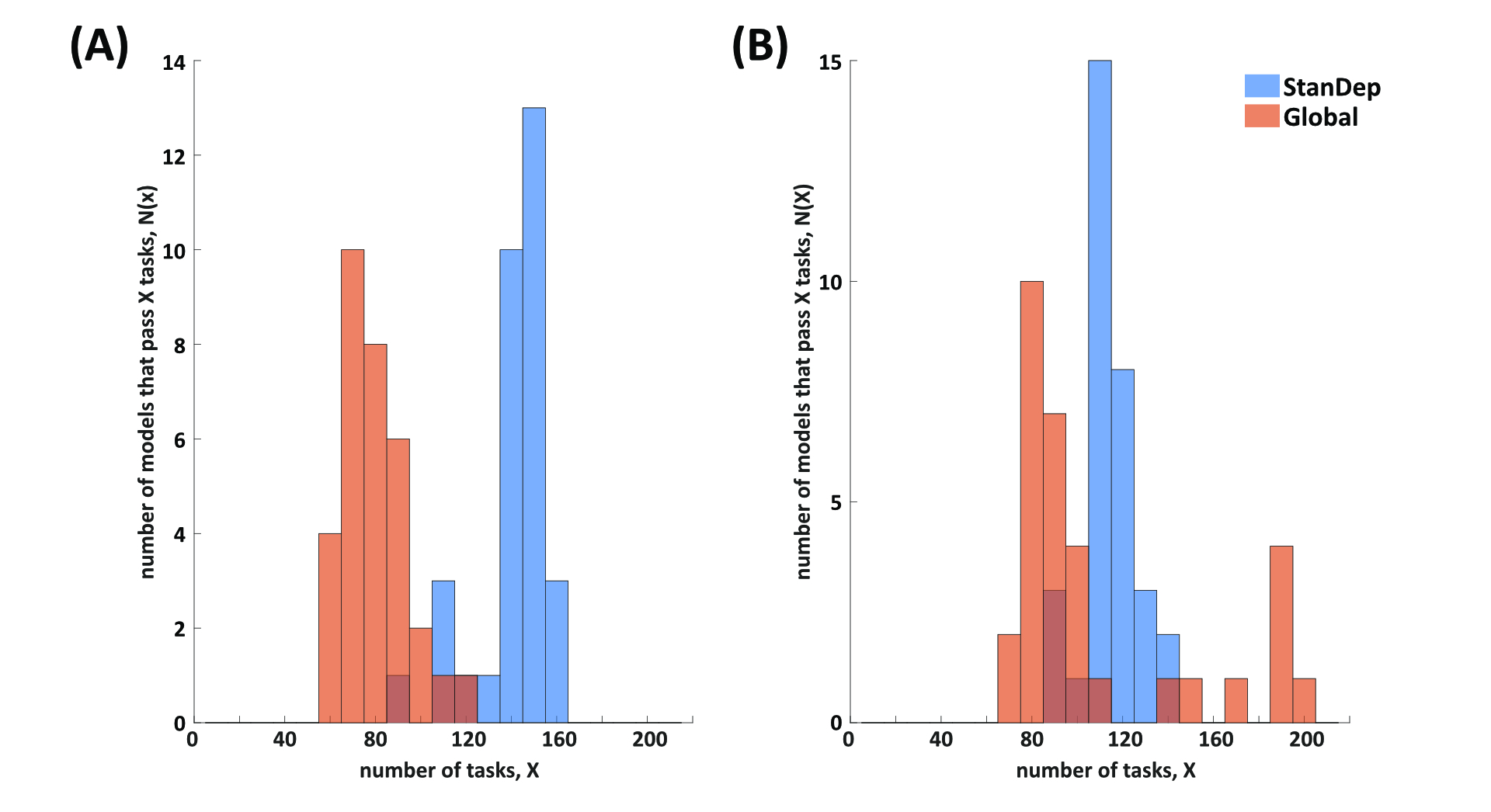

Supplement: S33 Fig — Distribution of metabolic tasks passed by models of human tissues extracted using (A) fastCORE and (B) mCADRE. StanDep models (blue) tend to pass more tasks than global models (red). (JPG) [file pcbi.1007764.s036.jpg]

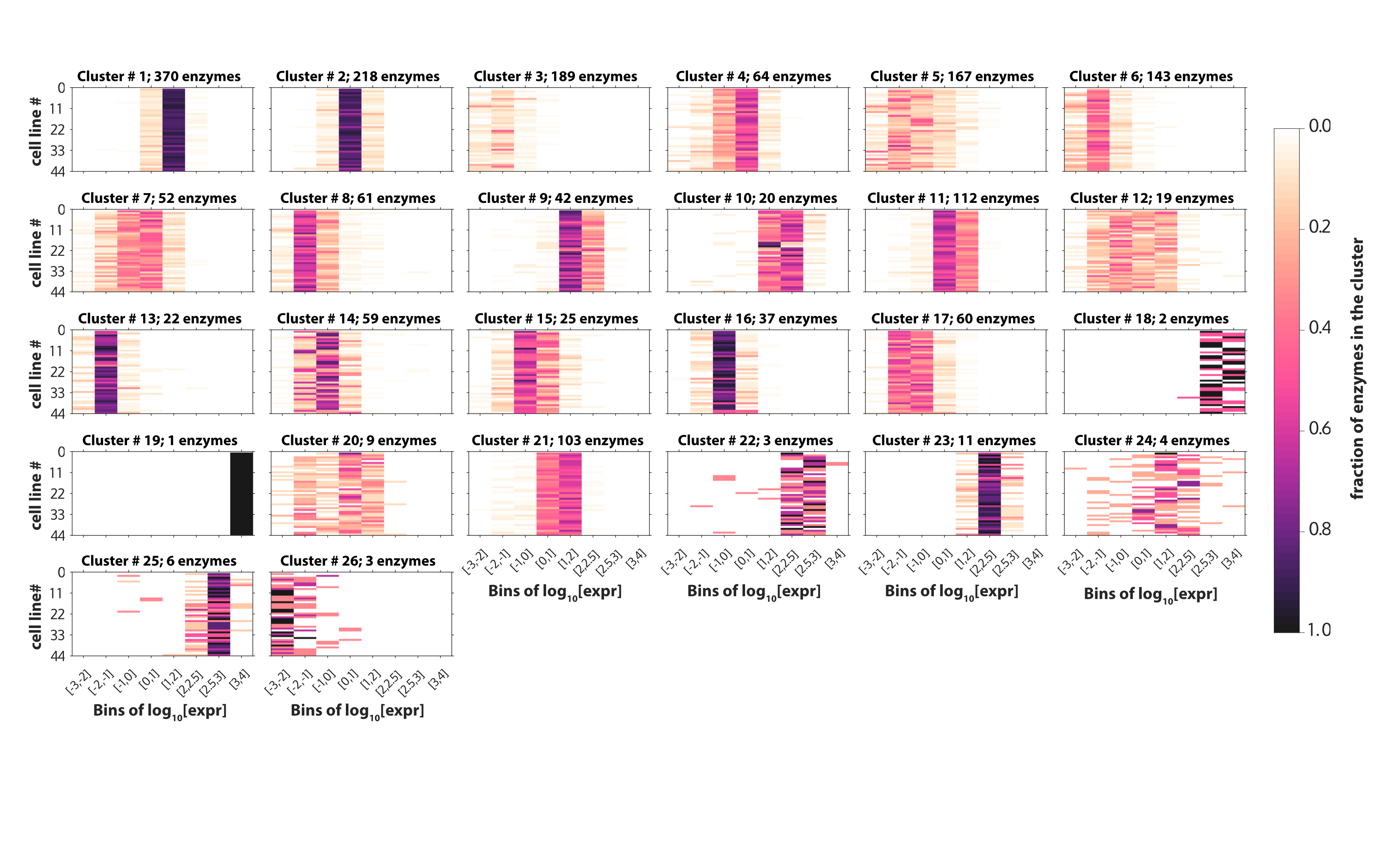

Supplement: S34 Fig — Heatmaps are shown for all 26 clusters. Black represents all the enzymes in that cluster are binned in a certain expression range for a given cell line. White represents none of the enzymes in that cluster binned in a certain expression range for a given cell line. (JPG) [file pcbi.1007764.s037.jpg]
