# Supplementary material for: StanDep: Capturing transcriptomic variability improves context-specific metabolic models
Source: PLoS Comput Biol. 2020 May 12;16(5):e1007764. doi: 10.1371/journal.pcbi.1007764 (PMC7244210; doi:10.1371/journal.pcbi.1007764)
Supplement: Attachment — Submitted filename: ResponseToReviewers.docx [file pcbi.1007764.s038.docx]

**Reviewer #1:**

Context-specific metabolic networks that integrate omics data with metabolic networks have been widely used to make important biological discoveries from microbes to human. One of the most important decisions in making context-specific models is defining a threshold above which a reaction is considered active. Despite the importance, the decision is often made somewhat arbitrarily and without much guidelines or consensus in the modeling field. The authors presented StanDep, a rigorous method that uses clustering to mitigate the arbitrariness in making this decision, potentially improving the robustness and consistency of context-specific models in the future. The authors did extensive benchmarking to demonstrate the performance of StanDep. StanDep is a new valuable tool for the metabolic network modeling community. However, this novel method and the manuscript itself might benefit from a few modifications suggested below.

Major:
StanDep included larger fraction of housekeeping reactions. But does it also included potentially false-positive reactions that should not be in the model?

This is an important point and we appreciate the reviewer’s suggestion to address it. Thus, we have added a paragraph in the discussion section to clarify that it is difficult to qualify a reaction to be a false positive reaction for a model. To explain this further, we would like to point to a case discussed for C. elegans, i.e. Peroxisomal fatty acid beta-oxidation pathway which was enriched in hypodermis, intestine, and rectum. While we were able to find studies showing the role of this pathway in hypodermis and intestine, we did not find any for rectum. This could be because the cell type hasn’t garnered enough interest within the community. Therefore, it is difficult to argue whether presence of a reaction is a novel discovery or false-positive.

Besides increasing the coverage of house-keeping genes/reactions, does StanDep improve models’ ability to simulate tissue-specific metabolic functions (e.g., liver metabolic tasks)?

We have performed metabolic task analysis of all 32 human tissues (fastCORE and mCADRE) and all cancer cell lines. I performed metabolic tasks analysis and found that StanDep-based models were able to pass more tasks than the global models for both mCADRE and fastCORE. We could not find a list of liver-specific metabolic task. Therefore, we simulated 210 human metabolic tasks that we recently published (Richelle et al., PLOS Computational Biology, 2019). Most of these tasks should occur in majority of tissues. We found that models derived using StanDep have higher coverage of these tasks. To address reviewer concern, we have added an additional excel file in the supplementary information (Supplementary Table 1) indicating the failure or success and a supplementary figure (Figure S36) showing that StanDep models tend to pass more metabolic tasks in each of the models.

In the StanDep-extracted models accurately capture essential genes, the authors seem to suggest that 58 genes that were accurately predicted by StanDep-derived models only are better supported by CRISPR loss of function screens. In addition to the high-level statistic, can specific examples be provided that the lethal genes uniquely captured by StanDep are biologically meaningful?

The gene essentiality is dependent on the structure of the extracted models, not only the core reaction lists but also the extraction methods. In Figure S5, we have discussed the models extracted using fastCORE. Therefore, we choose an example from the same comparison. As shown in Figure S5 (dotted blue line), some of the essential genes were accurate in nearly all of the NCI-60 cancer cell line StanDep-derived models but not in their localT2 counterparts. We found that these genes specifically belonged to oxidative phosphorylation. Specifically, these were subunits of cytochrome C oxidase (CYOOm3). Recently, oxidative phosphorylation has garnered interest for targeting cancer due to its important role in dissipation of excess electrons (Nayak A et al., Cancers, 2018). While the reaction itself was present in both set of models, only StanDep-derived models accurately identified them as essential. To address the reviewer’s concern, we have added the following sentences: “ Among these was also 6 subunits of cytochrome C oxidase, an enzyme belonging to oxidative phosphorylation. Recently, oxidative phosphorylation has garnered interest for targeting cancer due to its important role in dissipation of excess electrons [42]. While the reaction itself was present in both set of models, only StanDep-derived models accurately identified them as essential.”

Figure 2B-D are not clearly explained. What does the numbering on x-axis mean, how are they ordered, how is the p-value on y-axis calculated? The figure legend mentioned that “(C) Reactions which are captured by only StanDep mainly belong to low but tightly expression clusters.” However, there is no expression level information in Figure 2C.

We have changed figure captions for Fig 2.

Minor:
MEM was first used on page 5, line 138 in the main text without fully spelling it out as model extraction method. Although it is spelled out in Figure 1 caption, it still helps reading if it is also described in main text as well.

Added MEM with model extraction method to ensure readability.

Self-consistent is not quantitatively defined in page 5, line 139-142. Does higher self-consistency mean that the core reactions defined by StanDep require less non-core reactions to support?

Added “(i.e. MEMs add fewer unsupported reactions)” where the reviewer mentions it.

In figure legends, HK is used as an acronym for housekeeping. To some people, HK is often an acronym for hexokinase, a well-known, classical metabolic reaction, causing potential confusion.

Expanded HK to housekeeping in figure 2 and 3

**Reviewer #2**

The paper of Joshi et al. deals with an issue arising during the generation of context-specific metabolic networks based on transcriptomics data. There are already several types of algorithms (reviewed and categorized in reference [45]) which can be used for context-specific metabolic networks, and majority of them require setting a threshold value for declaring a reaction active. This threshold is either set to hold for all genes or is gene-specific. The authors propose a heuristic, StanDep, that takes a middle road, whereby the threshold value is determined per groups of genes. The extracted models from different scenarios (i.e. conditions and organisms) are partially tested against lists of housekeeping genes, CRISPR data, and RNAi phenotypic data. Therefore, the main contribution is the way to set the threshold, from which point the study depends on existing constraint-based approaches for context-specific network extractions.
I find that this a small contribution which also suffers from additional issues, which I detail below:
1. The author claim that the thresholding method involves: (1) cluster distribution of individual gene expression, considering multimeric and isozyme relationships (line 86). Is there support that the clusters contain protein complexes and / or isozymes. To the best of my knowledge, co-expression / transcript similarity can partly explain protein complexes, but only if complex stoichiometry is factored in; in addition, this rarely holds for isozymes. The authors should provide evidence for this claim.

We thank the reviewer for pointing this out. Perhaps, our explanation was not clear. The conversion of gene expression data into “enzyme abundance” is a standard procedure. This is accomplished in two steps: (1) minimum of the gene activity is chosen for multimeric enzyme complexes, and (2) the maximum or sum of the isoenzymes are taken to resolve multiple enzymes being able to catalyze a reaction. However, in our case, we have not resolved the isoenzymic relationships but left them as is (i.e. a reaction can still have multiple enzymes which may end up in different clusters and continue to prevail in the extracted model). Besides this, we actually showed that the clusters are also enriched in housekeeping reactions (i.e. enzymes which are responsible for performing housekeeping functions); thus, suggesting that clusters contain some meaning.

The reviewer also points out that complex stoichiometry is an important issue and we have not considered that. We agree with this and have therefore, added the following in the methods section “Data processing”: enzyme complex stoichiometry was not considered as it is not fully understood for all the enzymes involved (Christoph Juschke et al., Genome Biology, 2013).

2. The first step in the threshold selection requires hierarchical clustering based on Euclidean distances between gene expression profiles. While it is obvious that magnitude of gene expression is taken into consideration by following this approach, the reasons for not using other approaches (results shown in Supplementary Figures), are not provided. The results in the Supplementary Figures must be critically discussed in the main text—for instance, why is the comparison done for 26 clusters? Do all similarity measures yield the same number of clusters when following the procedure described in the Methods?

Given that we use only Recon 2.2 throughout the manuscript, the only variable in our setup for extraction of models is the core reaction lists. The clustering here is used as a mathematical device to split up genes into smaller groups. Therefore, if there is high similarity between core reaction lists, the extracted models will also be highly similar. The comparison was done for only 26 clusters as they showed high similarity in core reaction lists even upon further increasing the clusters. All distance measures may not yield the same number of clusters. Further, not all distance measures should be applicable as some are only pertinent to continuous data while others to discrete/categorical data. However, for 26 clusters, we showed that the Jaccard similarity for core reaction lists is nearly 90%. This suggests that there would be little difference in the models obtained using core reaction lists generated from either distance measures. This high similarity is because of the large number of clusters (26) chosen. By choosing a sufficiently large number of clusters, we minimize the effect of number of clusters on the extracted models.

3. The approach for selecting the number of clusters does not correspond to the steps described in the Methods. Lines 313 and 314 state that “the number of clusters is determined such that all pathway[s] is enriched in at least one cluster”. This is a—major—concern for three reasons: (1) it is difficult to imagine how this condition can be ensured, and if so, that this results in a single number of clusters, (2) the logic for selection of thresholds is then circular, since every pathway will get respective thresholds, thus partly ensuring its inclusion of the context specific model. The bottom line is that the selection of thresholds—is not—based only on the transcriptomics data, as claimed, but also includes the model structure (i.e. pathway information), and (3) Jaccard distance of core reactions, mentioned on lines 91-94, is not used for selection of thresholds, but for comparison of the resulting sets of core reactions. The authors must resolve the circular argument, since the results obtained are no surprise and represent artefact of the threshold selection approach, and account for the other noted discrepancies.

We thank the reviewer for bringing this to our attention and we understand the reviewer’s concern. The approach for selecting the number of clusters is discussed in the results section “Preprocessing transcriptomics data using StanDep”. The number of clusters were chosen such that any further increase in the number of clusters results in large Jaccard similarity (>90%) in core reaction lists. The section mentioned by the reviewer was therefore removed and corrected version was fixed in the methods section “Deciding number of clusters” as well. We apologize for the confusion.

4. The approach for replacing the biomass reactions must be detailed. The reason is that the authors already comment that forcing flux through the biomass reaction “rescues” some core reactions from central carbon metabolism.

We thank the reviewer for this question. The biomass reaction has been explained in detail in our previously published paper (Opdam et al., Cell Systems, 2016), same methodology was used here as well. We have therefore, added “..and has been discussed in our previous publications [13,25]”.

5. Figure S1 cannot be discussed without having access to the size of the models, which are not presented in the present manuscript. Going over the procedure for threshold generation, it seems that the larger number of HK genes may be due to the fact that the extracted models are themselves larger? Another reason why I believe this to be the case is that the comparisons in Fig. 3 A and B are based on fractions, and not the number of reactions!

In reference to Figure S1in the manuscript, we haven’t discussed extraction of models yet. Therefore, may add confusion for the readers. In Figure S1, we are discussing the length of the core reaction lists, which are completely independent of the extracted models. In fact, it is the extracted models that dependent on the core reaction lists as they are one of the inputs for the MEMs. The procedure for extraction of models is shown in Figure 1. Figure S1 demonstrates that core reactions derived using StanDep are larger than those derived using other thresholding methods. Indeed, larger core reaction lists are likely to generate larger extracted models. It should be noted that we also show in Fig S7 that MEMs added fewer fraction of reactions when core reaction lists belonged to StanDep. Therefore, keeping these things in mind, larger number of housekeeping genes aren’t due to increased model size but due to increased core reaction lists which are calculated using StanDep as shown in Figure S1. Thus, StanDep is responsible for increased coverage of housekeeping reactions in the extracted models. We also show in Figure S8 the ratio of number of reactions in StanDep to LocalT2. As Figure S8 shows, most of the fastCORE, MBA, and mCADRE models are of similar sizes. Larger models do occur but mostly when GIMME, iMAT, and INIT are used. In Figure 3A-B, we have normalized the numbers with the maximum housekeeping genes present in the flux-consistent genome scale model of Recon 2.2 (317 housekeeping genes and 929 housekeeping reactions). To address reviewer’s concern, we have added the normalizing numbers for housekeeping genes and reactions in the Figure 3 caption.

6. Line 151 – 152 “Overall, we found that StanDep contained more housekeeping reactions than localT2 models (Fig. 3A&B).” –does not—reflect what is shown in the figure panels! These figure compare fractions, which is not the same as numbers. In addition, a larger fraction of HK reactions was found only for mCADRE, MBA, and GIMME (and only MBA and GIMME with respect to HK genes).

As discussed in reviewer concern above, the normalization was done using a constant number and therefore does reflect the statements made in the manuscript. To address the reviewer concern, we have added the normalizing numbers for housekeeping genes and reactions in the Figure 3 caption.

7. The higher predictability / accuracy of predictions for essential genes, based on inspection of Fig. S17, is not justified. Further, I do not see what the further grouping of predictions (paragraph on lines 192-200) brings to the performance of the proposed heuristic.

Figure S17 is the violin plot of actual data of total predictions, accurate predictions, and % accurate predictions of gene essentiality. The statistical test (left tailed F-test) comparison of that figure is reported in Figure 3D which clearly shows statistical significance (p < 0.05) for number of predictions and number of accurate predictions. Further, Figure 3D is a test for difference in variance rather than higher accuracy. As clearly shown by figure 3D, accuracy (orange bars) was not statistically significant for any of the extraction methods. Therefore, we are claiming that StanDep is precise without sacrificing accuracy of the models when simulating single gene deletions. This means that if two models are built using StanDep, they both have similar accuracy. This is not true for the models built using existing methods; two models built using existing methods can vary significantly in their accuracy. Thus, making StanDep more reliable than existing methods. We could remove the reference to Figure S17 if the reviewer would like us to.

We thank the reviewer for pointing this out. The paragraph on lines 192-200 shows that models built using StanDep are not only capable of capturing features specific to all contexts of an organism (such as housekeeping genes) but also genes which are specifically essential in fewer or even one cell line. We have added the following sentence to the paragraph: “These results suggest that StanDep-derived models contain both: ubiquitously essential genes (22.12%), and cell line-specific essential genes (26.54%). Both of these are important determinant of quality of models.”

8. The authors talk about “enzyme expression” and “enzyme-specific variability”, which is technically wrong. These can be misinterpreted to mean “abundance of enzyme”, which is not available in transcriptomics data (but only in quantitative proteomics data). This can be corrected by writing “expression of enzyme-coding genes” and “variability of enzyme-coding genes”.

We thank the reviewer for pointing this out and have fixed it accordingly to avoid any confusion. In the discussion section where the term was first used in the main manuscript, we have added “(i.e. combined expression of enzyme-coding genes involved in a reaction)” and in the methods section where the term was first used in the main manuscript, we have added following sentences: “Please note that we do not mean “abundance of enzyme”. Here on we use the term “enzyme expression” to describe combined expression of enzyme-coding genes for a reaction.” These changes were also made to Figure 1 captions.

Minor
9. LocalT1, LocalT2, and global should be described for completeness in the main methods, rather than in the supplementary materials.

We have included these are part of main methods section.

10. The authors should comment about the reasons for excluding threshold-independent approaches for context-specific network generation.

We understand the reviewer comment. Therefore, we have added the following line in the introduction section: “A method that uses differential expression analysis to identify the core reaction set [23] will eliminate the arbitrariness involved in selection of thresholds; but will fail to capture such housekeeping reactions as it removes such features from the data.”

**Reviewer #3**

In this manuscript, the authors developed a heuristic method to support the existing model reduction algorithms to prune the genome-scale models to build context-specific metabolic models. The new method takes into account expression pattern across different contexts and determines thresholds based on the hierarchical clustering compared to an arbitrary threshold used in the current methods. The developed method can be useful to improve the quality of context-specific models built from human genome-scale metabolic models; however, such evaluation was only tested purely based on inclusion of housekeeping reactions and gene essentiality. Furthermore, the results are not presented consistently with too many details leaving to the Supplementary material.
Major concerns:
-There are other model reduction methods in the literature that do not use any arbitrary cut off values (e.g., Jensen and Papin, Bioinformatics 2011), so authors need to establish first why to use thresholds after all?

The example stated by the reviewer uses differential expression only. However, including only the differential expression may not guarantee the presence of constitutively expressed or housekeeping functionalities. We have shown in this study that inclusion of such reactions could only be insured by their inclusion in the core reaction lists (i.e. reaction lists calculated after applying cut off values). Further, penultimate line in the second paragraph states that to integrate transcriptomics data, one would have to make a call on whether enzyme-coding genes responsible for the metabolic reactions are expressed. Please see, “.threshold parameter – genes expressed above the defined threshold are interpreted to be active and part of the ‘core’.” To address this, we have added the following lines to the paragraph in introduction: “A method that uses differential expression analysis to identify the core reaction set [23] will eliminate the arbitrariness involved in selection of thresholds; but will fail to capture such housekeeping reactions as it removes such features from the data.”

-It is evident from their analysis that the previous methods were not capable of capturing many reactions belonging to housekeeping genes indicating the poor quality of the generated networks. Even with StanDep, mean fraction of housekeeping reactions included in core reaction lists was 0.80, indicating many more housekeeping reactions are still missing in the final models. Therefore, authors need to check the quality of the generated context-specific models based on some functional evaluations together with gene essentiality.

We thank the reviewer for bringing this point. In the manuscript, we have analyzed and compared our models to models built using existing methods. We agree that there are still many housekeeping reactions that are still not captured in the core reaction list as indicated by Table 1. However, we would also like to bring reviewer’s attention to Figure 3B. The plot clearly shows that for MBA-like methods, the coverage of housekeeping reactions was higher than 0.8. While the increase in number of housekeeping reactions was significant for models built using mCADRE, MBA and GIMME; the coverage of housekeeping reactions did not decrease for other methods. Similarly, coverage of housekeeping reactions in models of human tissues was also around 90% (Figure S9). Thus, we don’t think the quality of the models is in the question. Further, given about 90% coverage of housekeeping reactions in NCI-60 cancer cell line and tissue models, we did perform gene essentiality analysis for these models and show that accuracy of the StanDep-derived models was not less than the models built using existing methods (Figure 3D and Figure S17).

-The authors claim development of hundreds of models for the NCI-60 cancer cell lines that increased inclusion of housekeeping genes. It would be helpful for the reader if you exemplify application of one such model and show how StanDep makes a difference in predicting the outcomes compared to a model developed with arbitrary thresholds.

The application of context-specific models has been discussed in the first paragraph of the introduction. In the manuscript, we gave examples of improvements over arbitrary thresholds. If a gene/reaction is not present in the model, the model cannot be used to test perturbations of this gene/reaction. Specifically, we discussed the Biotin metabolism and Glycan metabolism case and the importance of housekeeping reactions in these pathways to human cells.

-The supplementary section contains too many figures, not referred consistently in the main text. Some of those supplementary figures depicting important findings should be in the main text for easy reference to the reader.

We are happy to see that the reviewer found the supplementary figures useful for the main text. We plan to finalize the numbering for the supplementary figures upon acceptance. Also, if the reviewers would like to see certain figures moved from the supplementary information to the main text. Please do let us know and we would gladly do so.

- The authors should condense the Supplementary material by reducing the unnecessary figures that are not adding value to the main text.

While we agree that there are a lot of supplementary figures, we feel that the figures we provide are important for full disclosure. Since many will only be needed for specific readers who may want to implement the method and understand its nuances, we have left these in the supplementary material and tried to make the supplementary material easier to navigate. We would happy to make further adjustments as needed.

-As mentioned in the Discussion, the method requires availability of large sets of gene expression data to define thresholds. Therefore, the authors should mention what is the minimum amount of data required to use the method. They should also mention other limitations of their proposed method, if any.

We think these are the main limitations: Availability of large dataset and that the samples in the dataset should be sufficiently diverse such as that tissues, cancer cell lines, or cell types. For example, if the dataset is of a particular cancer cell line with single genes mutated, StanDep may not be able capture such small diversity within the samples or may need significant modifications to integrate such a transcriptomics dataset with the metabolic model. We have mentioned both these points in the discussion section. If the reviewer has any other types of dataset, we would be happy to include them in the discussion.
