# Supplementary material for: StanDep: Capturing transcriptomic variability improves context-specific metabolic models
Source: PLoS Comput Biol. 2020 May 12;16(5):e1007764. doi: 10.1371/journal.pcbi.1007764 (PMC7244210; doi:10.1371/journal.pcbi.1007764)
Supplement: Attachment — Submitted filename: ResponseToReviewers.docx [file pcbi.1007764.s039.docx]

**Reviewer #1**

The authors have adequately addressed my previous concerns. One further suggestion is that all supplemental figures should be mentioned somewhere (main text, Method, or supplemental notes), so that readers have better context when navigating them.

We thank the reviewer for taking the time to read our manuscript and giving us valuable feedback to improve our manuscript. Besides making sure that all supplementary figures are mentioned, we have also fixed the supplementary figure numbering.

**Reviewer #2**

Review of the revised version of “StanDep: capturing transcriptomic variability improves context-specific metabolic models”
I would like to thank the authors for attempting to consider all points I raised on the first version of the manuscript. Nevertheless, I find that the revised version still contains some pressing issues which need further explanations. Meanwhile, I found additional issues with the figures in the main text, which I detail in my comments bellow:

1. The authors state that they resolved AND GPR rules, but did not consider OR GPR rules. Integration of transcriptomics data with both rules is well established, as the authors state in their response, so I do not see the reason for neglecting the consideration of OR rules. This must be addressed as the decision seems arbitrary.

We thank the authors for bringing this to our attention. We realize how what we have done may seem like an arbitrary choice. In fact, we have rather removed the arbitrariness by relaxing the assumption that each reaction can only be catalyzed by one enzyme.

2. The presentation of the approach to arrive at the thresholds based on the different clusters is greatly improved. However, what struck my curiosity is that if the respective thresholds \Theta_c is 100%, then the mean of the data set is used. In how many cases in the analysis did this occur? Again, this sounds like an arbitrary choice!

Mathematically, a threshold of 100 %-ile for a cluster indicates that all the genes are OFF. This would happen for only one cluster. For our analysis, it happened for cluster 26 which contained 3/1802 enzymes. Yes, it could be an arbitrary choice but we don’t think it will have much of an effect. These genes could be important and require a separate investigation. However, it is difficult to them justice here. Our study here is only trying to show that variability is an important factor in making context-specific models, how one may include variability in making context-specific models, and what are its consequences on the extracted model.

3. Another point to arbitrariness is the way histograms are created – the number of bins used will certainly affect the derived distance measures. The authors should detail some well-established rules from data science used to address this point.

As far as we are aware, how transcriptomics data should be binned is still an open question. Of course, for this, one would also have to know how much sensitivity there is within the dataset, i.e. how often a gene-activity is off at 9 TPM but on at 10 TPM and so on. StanDep, as a method, will benefit hugely from knowing answers to such questions. As far as distance measures are concerned, if one understands the data better, one can construct a novel distance metric too. Again, we would like to point out that clustering here is used as a mathematical device that can capture heuristics, for e.g. clusters enriched in housekeeping genes/reactions. The method should be seen as groundwork for creating models that acknowledge the diversity in gene expression distribution patterns.

4. I still do not agree with the justification regarding the number of clusters used. This, too, is an arbitrary choice, since the > 90% similarity is an arbitrary choice. (I do accept the argument about the subsequent Jaccard comparison, to justify the usage of a particular distance measure).

As we tried to explain in the previous revisions, the similarity between any two given extracted models would only be more than the similarity between the corresponding core reaction lists. We show this in the figure below as points lie in lower half of the X=Y line.

Therefore, if there is a similarity of > 90% in the core reaction lists, then the corresponding models are likely to be even more similar, i.e. have even higher Jaccard similarity. A Jaccard similarity of >90% indicates that >95% of the two core reaction lists are identical. One may go for higher number of clusters, but it doesn’t give us a huge change in the core reaction list, i.e. it may not give us a huge change in the models too.

This being said, of course, one could find other justification for choosing the number of clusters. In fact, there are many; for example using Silhouette values. It also depends on for what one is going to be choosing the clusters. Further, different metrics may suggest different number of clusters.

5. The authors should detail the replacing of the biomass reaction, since the methods section should suffice to understand what was done in the present study.

We added the line “The average composition of these components were determined from literature [49–59]. The stoichiometric coefficients of the metabolites in the biomass equation are presented in Supplementary Table 2. Please see Methods of Opdam et al. 2017 for how these stoichiometric coefficients were calculated [13].”

6. The performance of the approach, other than the inclusion of more housekeeping genes (Table 1 and Fig. 3), does not truly provide further conclusive arguments that they improve context-specific models. The authors write that with respect to essentiality they do not perform worse than the other approaches. Hence, more tests are needed to justify the title and this major claim of the manuscript.

Indeed, we agree with the reviewer. It is important to have a high coverage of housekeeping genes in the model. Housekeeping genes are important for capturing the organismal context, i.e. they are markers that identify the model belonging to the same organism as the data. While the models do not perform worse on accuracy, they perform very well on precision. By precision, we mean that models prepared using StanDep have similar accuracy than those prepared using LocalT2. This is clearer in Figure S17B; the distribution of accuracy is wider (in some cases, bimodal) for localT2-based models (purple) than it is for StanDep-based models (orange). The statistical test for this statement is shown in Figure 3D. The advantages of precision lie in the fact that performance of the models prepared using our method is predictable and reliable. Thus, this is a valuable attribute for a method to have.

7. The authors did not cast their net sufficiently wide to find out that there are other threshold-free approaches for integration of transcriptomics data, other than the one cited. For instance, Robaina-Estevez et al. published and used RegrEx in several situations; comparison with this approach is also desired.

Indeed, it would make for a great study to compare threshold-based versus threshold-free transcriptomic data integration methods. However, for that to happen one would have to decide on the best thresholding method which has been applied by other groups to other datasets. Following this, one would have to also decide on the threshold-based model extraction method (only 6 of which are discussed here, there are more). It should also be noted that there are a few threshold-free approaches for integration too. This alone leads to many different combinations to compare RegrEx with. Such a study can be valuable in design of methods to extract context-specific models. However, the point of this paper was directed towards thresholding-based extraction methods and benchmarking StanDep with existing thresholding methods and not to qualify a threshold-based or threshold-free extraction method good. Further, given the unanswered questions in the field that we mentioned, we think it wouldn’t be fair to compare StanDep (the only paper on it is being reviewed here) with RegrEx. However, we agree it could make for a great follow up study.

8. The number of supplementary items is staggering. I did attempt to consider all of the tables and figures, but I chose to comment only on the figures of the main text (see next point). The authors may consider removing items which are not truly necessary.

We are glad that the reviewer finds our supplementary items in surplus. However, we think the extra figures/tables/texts help in understanding of our method for people who would like to reproduce/understand/apply our method to their own dataset. This is evident from the fact that we have added more supplementary figure to accommodate the suggestions made by another reviewer. As the figures are in supplementary information, we don’t see why this is an issue.

9. In figure 1, it is desirable to somehow specify that distributions are clustered, rather than gene expression profiles (this may be apparent from the y-axis label, but it could be more explicitly stated). Figure 2 A, x-axis label makes no sense; Figure 2B, x-axis label has issues; Figure 3 C – check the % in the pie charts, some do not correspond to the visuals. Figure 3D is very difficult to follow: what are total predictions? Accurate predictions with respect to what? accuracy in % (figure panels should be self-explanatory and understandable based on the caption provided.)

In Figure 1, we have added the line “distribution of gene expression are clustered.” In Figure 2A, x-axis refers to the what fraction of the cluster are housekeeping reactions. In Figure 2B, the x-axis label was changed to “number of housekeeping reactions in StanDep but not in localT2.” In Figure 3C, we fixed the top pie chart to accurate labels. In Figure 3D, we have changed the legend as well as the caption.

10. I find that one example in Fig. 3C is too little a justification for the success of the presented approach.

In fact, we have discussed two examples: (i) Biotin metabolism in Fig 3C, and (ii) Phosphatidylinositol phosphate metabolism in Figure S14. Further, to show that these are not the only two, a detailed list of pathways where the differentially present housekeeping genes in StanDep models are shown in Fig S11 and Fig S12. Therefore, Fig. 3C and Fig S14 are only two examples out of the many hits shown in Fig S11 and S12.

Reviewer #3: No further comments

We thank the reviewer for their valuable input which led to improving the state of manuscript.
